# Does prior vaccination affect the immune response to seasonal influenza vaccination among older adults? Findings from a prospective cohort study in a Northeastern Province of Thailand

**DOI:** 10.1371/journal.pone.0279962

**Published:** 2023-02-03

**Authors:** Prabda Praphasiri, Kriengkrai Prasert, Manash Shrestha, Darunee Ditsungnoen, Malinee Chittaganpich, Sutthinan Chawalchitiporn, Fatimah S. Dawood, Supakit Sirilak, Joshua A. Mott

**Affiliations:** 1 Influenza Program, Thailand MOPH-US CDC Collaboration, Nonthaburi, Thailand; 2 Nakhon Phanom Provincial Hospital, Nakhon Phanom, Thailand; 3 Faculty of Social Sciences and Humanities, Mahidol University, Nakhon Pathom, Thailand; 4 National Institute of Health, Thai Ministry of Public Health, Nonthaburi, Thailand; 5 Influenza Division, US Centers for Disease Control and Prevention, Atlanta, GA, United States of America; 6 Office of The Permanent Secretary, Ministry of Public Health, Nonthaburi, Thailand; IAVI, UNITED STATES

## Abstract

**Background:**

We measured the immunogenicity of seasonal trivalent inactivated influenza vaccines (IIV3) among older Thai adults and the effect of one-year prior vaccination status on immune responses.

**Method:**

Adults aged ≥65 years (n = 370) were vaccinated with Southern Hemisphere IIV3 in 2015. Hemagglutination inhibition assays were performed using goose red blood cells on sera collected from the participants at baseline and after 1, 6, and 12 months of vaccination. Prior year vaccination (in 2014) was verified with the national health security office database. We analyzed the associations between prior vaccination and geometric mean titers (GMT) at each time point using generalized linear regression on logged transformed titers, and seroprotection and seroconversion using Log-binomial regression.

**Results:**

At baseline, previously vaccinated participants (n = 203) had a significantly higher GMT and seroprotection against all three influenza strains than those previously unvaccinated (n = 167) (all p-values <0.001). Seroprotection rates were similar after one month in both groups for A(H1N1)pdm09 (adjusted risk ratio [aRR] 1.10, 95% CI 0.97–1.25), and A(H3N2) (aRR 1.08, 95% CI 0.87–1.33), but higher in previously vaccinated persons for B (aRR 1.20, 95% CI 1.08–1.32). At 12 months, 50% or more had seroprotection in previously vaccinated group with no difference between previously vaccinated or unvaccinated persons. Seroconversion was lower in the previously vaccinated group for A(H1N1)pdm09 (aRR 0.62, 95% CI 0.43–0.89), but did not differ between the two groups for A(H3N2) (aRR 0.94, 95% CI 0.69–1.28) and B (aRR 0.85, 95% CI 0.60–1.20).

**Conclusion:**

Influenza vaccination elicited good humoral response in older Thai adults. While seroconversion seemed attenuated in persons previously vaccinated for influenza A(H1N1)pdm09 (the only vaccine strain not to change), this was not apparent for influenza A(H3N2) and B, and prior vaccination was not associated with any inhibition in seroprotection.

## Introduction

Older adults are at a disproportionately higher risk of influenza-associated morbidity and mortality than their younger counterparts [1,2]. Although seasonal trivalent influenza vaccine (IIV3) is generally effective in preventing influenza and its complications [1], its effectiveness can vary according to season, depending on the match of vaccine strains to the circulating virus strains, and may reduce with increasing age [3,4]. In older adults, age-related changes can gradually reduce the capacity to develop vaccine-induced immunity through a process known as immunosenescence [5,6]. However, the results of immunogenicity studies, which test the antibody response of influenza vaccines by quantifying the amount of antibody seroconversion and duration of humoral immune response after influenza vaccination, have not been consistent among older adults [7].

A literature review found that adults aged 59 years or older had 2–4 times lower rates of seroconversion compared with younger adults [8]. In addition, different studies have reported intra-seasonal waning of immunity induced by IIV3 among older adults [9], prompting the use of higher doses and adjuvanted influenza vaccines to boost the immune response [10–12]. On the contrary, some studies suggest that the rapid decline of antibody response among elderly individuals within months of receiving influenza vaccine may not be correlated with age [13], but instead, linked with prior influenza vaccinations and higher levels of preexisting serum hemagglutination inhibition (HI) antibodies [14–17]. Therefore, confounding between age and prior vaccinations could explain the lower serological response to vaccination in older adults [18].

In Thailand, influenza transmission occurs year-round and is an important cause of morbidity and mortality resulting in substantial economic losses valued at approximately US$ 23.4–62.9 million annually [19,20]. During 2005–2008, an annual average of 36,400 influenza-associated hospitalizations and 300 deaths occurred, with significantly higher mortality rates in children and the elderly [21]. Owing to this burden of influenza, Thai adults aged ≥65 years have been recommended for annual vaccination with IIV3 by the Ministry of Public Health since 2008 along with seven other high-risk groups [22], and studies in Thailand have reported the effectiveness of IIV3 to be in the range of 47–56% in this age group [23–26]. Clinical trials have found significantly higher seroprotection rates against influenza virus strains among vaccinated Thai older adults than in the placebo group [27], even though seroconversion rates have been modest [28]. However, little is known about the effect of prior influenza vaccination on immune responses elicited by subsequent vaccination among older Thai adults. To provide additional evidence to inform local vaccine policy, this cohort study was conducted to measure humoral immune responses to influenza vaccine among community-dwelling Thai older adults during 2015–2016. We assessed any modification of immune response patterns that may have been associated with the reported prior vaccination in 2014.

## Material and methods

### Study design and study population

This prospective longitudinal study was nested in a larger cohort study that measured the effectiveness of the trivalent inactivated influenza vaccine in community-dwelling persons aged 65 years and older in Nakhon Phanom, Thailand (Thai Clinical Trials Registry ID: TCTR20150516001) [26]. A total of 384 adults aged ≥65 years who visited sub-district health centers and district hospitals requesting vaccination against influenza in 2015 were enrolled in this study using a convenience sampling method. The participants were followed up for 12 months in the 2015–16 influenza season (from May 2015 through May 2016) in That Phanom and Plapak districts of Nakhon Phanom province in the Northeast of Thailand where they received annual influenza vaccination. The participants had to be residing in the community for at least a year and able to communicate with the study staff to be included in the study. Institutionalized persons, prior recipients of the vaccine during 2015 (but before the study), those with an acute medical condition, or a person with a history of a bleeding disorder, a known allergy to influenza vaccine or egg, or any contraindication to venipuncture were excluded from the study.

### Influenza vaccination

Influenza vaccination was provided free of charge via a national campaign at sub-district health centers and district hospitals in May 2015. The vaccine strain composition differed for influenza A(H3N2) and influenza B (Yamagata lineage) in 2014 and 2015, while it remained the same for A(H1N1)pdm09 (Table 1).

**Table 1:** Influenza vaccine strain composition of southern hemisphere vaccines in two seasons from 2014–2015 and 2015–2016.

Influenza virus sub-type	2014–2015	2015–2016
A(H1)	A/California/7/2009 (H1N1) pdm09-like virus	A/California/7/2009 (H1N1) pdm09-like virus
A(H3)	A/Texas/50/2012 (H3N2)-like virus	A/Switzerland/9715293/2013 (H3N2)-like virus
B	B/Massachusetts/2/2012-like virus	B/Phuket/3073/2013-like virus

### Data collection

Enrollment questionnaires were administered by trained health volunteers and study team members at the sub-district health centers and district hospitals. Vaccination in 2014 was verified with the National Health Security Office (NHSO) database. The participants were vaccinated with IIV3 in the 2015–16 season and blood samples (5ml) were collected from the participants at four intervals during the study period by the study nurses. The first blood sample was collected prior to the vaccination, while the 2^nd^, 3^rd^, and 4^th^ samples were collected at 1, 6, and 12 months thereafter. Blood samples were obtained by research nurses at the participant’s home and transported back to the district hospital in ice packs for those who were unable to travel to the health facility. At the district hospital, sera were separated from the whole blood samples and stored at -20° C until tested. The serum samples were sent every week to a laboratory at the Thai National Institutes of Health (NIH).

### Hemagglutination-inhibition assay

In the NIH laboratory, serum antibody titers were determined by hemagglutination inhibition (HI) assay using 0.5% goose red blood cells (RBC) according to the WHO standard protocol as described previously [29,30]. HI antibody titer was determined from the reciprocal of the highest serum dilution that could completely inhibit hemagglutination of the goose RBC.

### Serological outcomes

Seroprotection was defined as HI titers of 1:40 or greater; whereas everyone with a baseline HI titer ≥1:10 who had a four-fold increase in post-vaccination antibody titer, or who had a baseline HI titer <1:10 and had a post-vaccination antibody titer ≥1:40 were considered seroconverted against influenza. The geometric mean titer (GMT) was calculated by taking the antilog of the mean of logarithmically transformed HI titers.

### Sample size and statistical analysis

**Fig 1:**
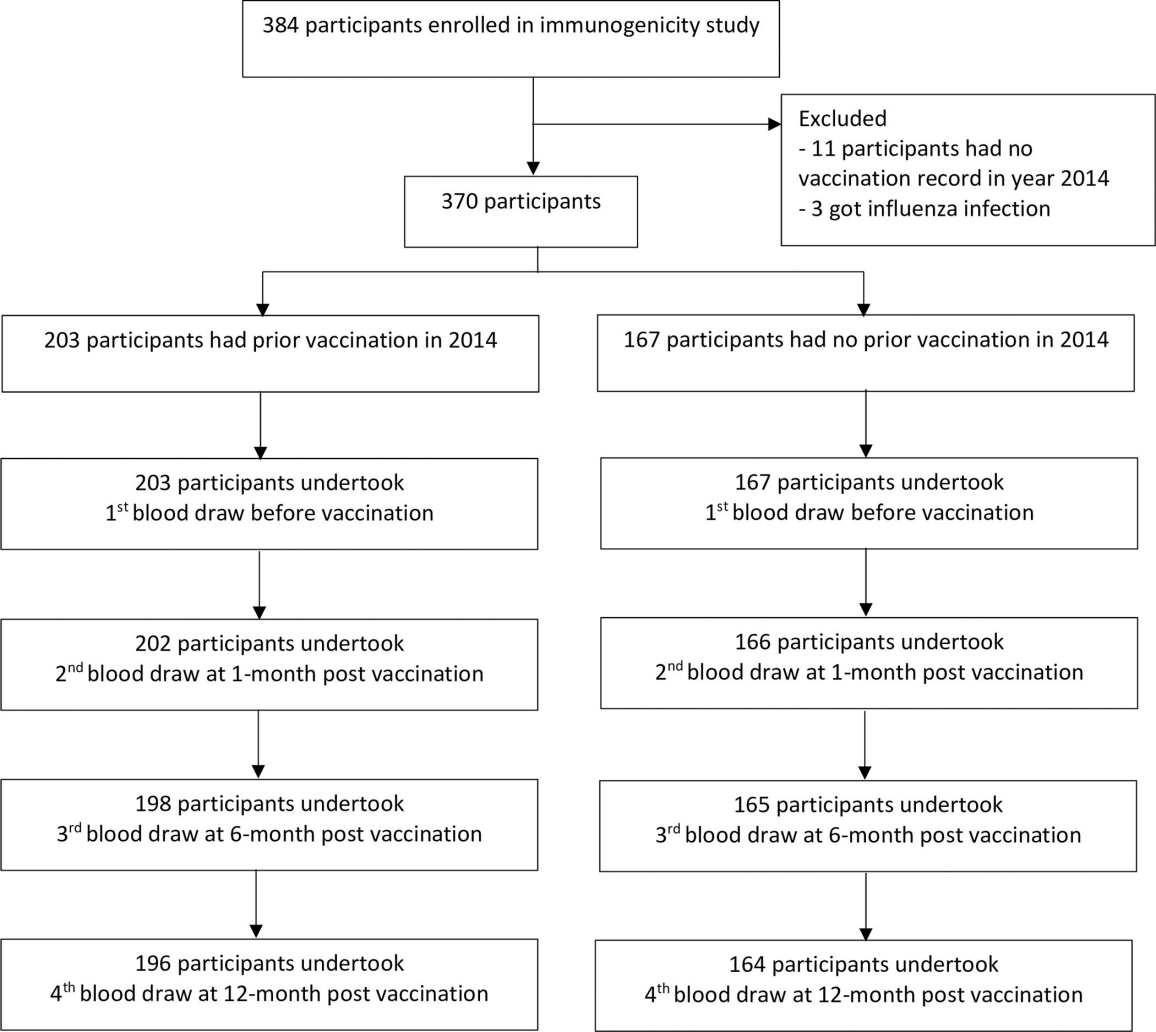
Enrollment and follow-up blood collection for immunogenicity study in adults aged 65 years and older Nakhon Phanom Province, Thailand, May 2015-May 2017.

A sample size of 384 was calculated in OpenEpi (www.OpenEpi.com) assuming a 50% seroconversion rate, the estimated size of the population of adults aged ≥65 years of the two districts to be 15,000, 5% type I error, and absolute precision of 5 percentage points. We excluded participants who had no vaccination record history in year 2014 (n = 11) and those who had an influenza virus infection by rRT-PCR-confirmed influenza (n = 3) during the study period. The final analytical sample comprised 370 participants by intention to treat (Fig 1). The 95% confidence intervals for GMTs were calculated from the Student’s t distribution of log10-transformed titers. We determined the association between 2014 vaccination and GMTs at each time point using generalized linear regression with an identity link function on logged transformed titers, adjusting for age group (<75 years/≥75 years) and sex. Estimates of seroprotection and seroconversion by vaccination history were also adjusted for age group and sex using Log-binomial regression adjusted for age group and sex. To determine the association of age on the duration of the immune response, we dichotomized age (<75 years/≥75 years) and assessed the difference in seroprotection rate at each time point, adjusting for sex and prior vaccination. Statistical analyses were performed using STATA software version 14.2 (StataCorp LP, College Station, TX, USA) and the significance was set at a p-value <0.05.

### Ethical considerations

After screening for eligibility by research nurses, the participants were provided explanation of the study objectives, possible risks, and the voluntary nature of their involvement in the study. Informed consent form was obtained prior to starting study procedures. In instances in which consenting persons were unable to read, the study staff read an oral consent script and then collected written documentation of oral consent for study participation. Consent forms were first written in English and then translated into Thai by an experienced translator, checked by a senior Thai scientist fluent in both Thai and English languages, and back translated to ensure accuracy. Approval for the study was obtained from the Ethical Review Committee, Department of Disease Control, Ministry of Public Health, Thailand (no. 4/58-743).

## Results

### Participant characteristics

We recruited 384 participants and excluded 11 due to no vaccination records, and 3 who had influenza infection during a study period. Of 370 enrolled participants, 203 had been vaccinated in 2014, and 167 had not. Of these, 196 participants who had been vaccinated and 164 who had not been vaccinated completed all four blood draws (Fig 1).

The median age of the participants was 71 years (interquartile range 68–76), and 63% of them were female. More than half of the participants were vaccinated against influenza in 2014 (203; 54%). 255 (69%) participants less than 75 years of age were more likely to have been vaccinated in 2014 than those 115 (31%) aged 75 years or more (Fisher’s exact test, p = 0.003) but there was no difference in the 2014 vaccination status by sex (p = 0.448).

### Immunological response to influenza vaccination

Baseline GMTs for influenza A(H1N1)pdm09, A(H3N2), and B were 14.0 (12.4, 15.8), 23.3 (20.6, 26.3) and 14.3 (12.7, 16.1), respectively, which increased one month after vaccination to 68.2 (59.3, 78.7), 327.3 (282.1, 379.8), and 73.2 (64.9, 82.6), respectively (Table 2). Seroprotective antibody levels (HI titer ≥1:40) at baseline were found in 27.6% (23.1, 32.4) of participants to influenza A(H1N1)pdm09, 40.8% (35.8, 46.0) to A(H3N2), and 27.3% (22.8, 32.1) to B, which increased to 74.5% (69.7, 78.3), 94.0% (91.1, 96.3), and 81.5% (71.1, 85.1), respectively one month after vaccination (Table 2). The percentage of participants who seroconverted after vaccination was 50% (44.8, 55.2) for influenza A(H1N1)pdm09, 81.5% (77.1, 85.4) for A(H3N2), and 56.8% (51.6, 61.9) for influenza B.

**Table 2:** Overall GMT (geometric mean titer) seroprotection and seroconversion overtime by influenza vaccine strains.

Vaccine strain	GMT (95%CI)	Seroprotection (95%CI)	Seroconversion (95%CI)
A (H1N1)			
Month 0	14.0 (12.4, 15.8)	27.6 (23.1, 32.4)	-
Month 1	68.2 (59.3, 78.7)	74.5 (69.7, 78.3)	50.0 (44.8, 55.2)
Month 6	36.2 (32.1, 40.9)	56.8 (51.5, 61.9)	-
Month 12	26.0 (23.1, 29.3)	46.9 (41.7, 52.3)	-
A (H3N2)			
Month 0	23.3 (20.6, 26.3)	40.8 (35.8, 46.0)	-
Month 1	327.3 (282.1, 379.8)	94.0 (91.1, 96.2)	81.5 (77.1, 85.4)
Month 6	114.6 (100.7, 130.4)	85.7 (81.6, 89.1)	-
Month 12	78.9 (69.7, 89.3)	79.7 (75.2, 83.8)	-
B			
Month 0	14.3 (12.7, 16.1)	27.3 (22.8, 32.1)	-
Month 1	73.2 (64.9, 82.6)	81.3 (76.9, 85.1)	56.8 (51.6, 61.9)
Month 6	36.7 (32.9, 41.0)	60.1 (54.8, 65.1)	-
Month 12	28.7 (25.9, 31.9)	49.4 (44.2, 54.7)	-

Compared to participants aged <75 years, those aged ≥75 years had a similar proportion with seroprotective levels at baseline for influenza A(H1N1)pdm09 (adjusted risk ratio [aRR] 0.98, 95% CI 0.59–1.63), influenza A(H3N2) (aRR 1.32, 95% CI 0.87–2.00), positively what was observed for B (aRR 1.64, 95% CI 1.02–2.64) (Table 3). During the study period, seroprotection rates were similar between participants aged <75 years and ≥75 years, for all strains (Table 3).

**Table 3:** Prevalence of sero-protection (hemagglutination inhibition titer ≥ 1:40) among elderly age 75 years or older and those with age less than 75 years at study baseline, 1-month, 6-month and 12-month follow-up.

Vaccine strain	Sero-protection		Adjusted RR (95%CI)	p-value^α^
	Age ≥ 75 years (n = 115) n (%)	Age < 75 years (n = 255) n (%)		
A (H1N1)				
Month 0	28 (24.4)	74 (29.0)	0.98 (0.59, 1.63)	0.950
Month 1	81 (70.4)	193 (76.3)	0.94 (0.66, 1.32)	0.712
Month 6	61 (53.5)	145 (58.2)	0.93 (0.64, 1.35)	0.699
Month 12	47 (41.9)	122 (49.2)	0.87 (0.59, 1.31)	0.503
A (H3N2)				
Month 0	52 (45.2)	99 (38.8)	1.32 (0.87, 2.00)	0.185
Month 1	108 (93.9)	238 (94.1)	1.01 (0.73, 1.39)	0.950
Month 6	98 (86.0)	213 (85.5)	1.01 (0.73, 1.41)	0.951
Month 12	88 (78.6)	199 (80.2)	0.99 (0.70, 1.38)	0.933
B				
Month 0	39 (33.9)	62 (24.3)	1.64 (1.02, 2.64)	0.042
Month 1	98 (85.2)	201 (79.5)	1.09 (0.79, 1.53)	0.580
Month 6	79 (69.3)	139 (55.8)	1.29 (0.90, 1.85)	0.164
Month 12	64 (57.1)	114 (46.0)	1.28 (0.87, 1.88)	0.203

RR = relative risk. CI = confidence interval

^α^Log-binomial regression adjusted for age group sex. Significance level at < 0.05.

### Duration of humoral immune response

The GMTs increased and reached a peak at one-month post-vaccination for all three strains and then gradually decreased over time during the influenza season (Table 2). GMTs for influenza A(H1N1)pdm09, A(H3N2), and B were 14.0 (12.4, 15.8), 23.3 (20.6, 26.3) and 14.3 (12.7, 16.1), respectively. GMTs at 1 month, 6 months and 12 months after vaccination for influenza A(H1N1)pdm09 were 68.2 (59.3, 78.7), 36.2 (32.1, 40.9) and 26.0 (23.1, 29.3), respectively; for A(H3N2) GMTs were 327.3 (282.1, 379.8), 114.6 (100.7, 130.4) and 78.9 (69.7, 89.3),respectively; and for B GMTs 73.2 (64.9, 82.6), 36.7 (32.9, 40.1) and 28.7 (25.9, 31.9),respectively. The percentage of participants with seroprotective levels of HI titer against influenza were similar in all three strains: GMT peaked at month 1 and then decrease overtime (Table 2).

### Effect of prior vaccination on

#### a) GMT

**Fig 2:**
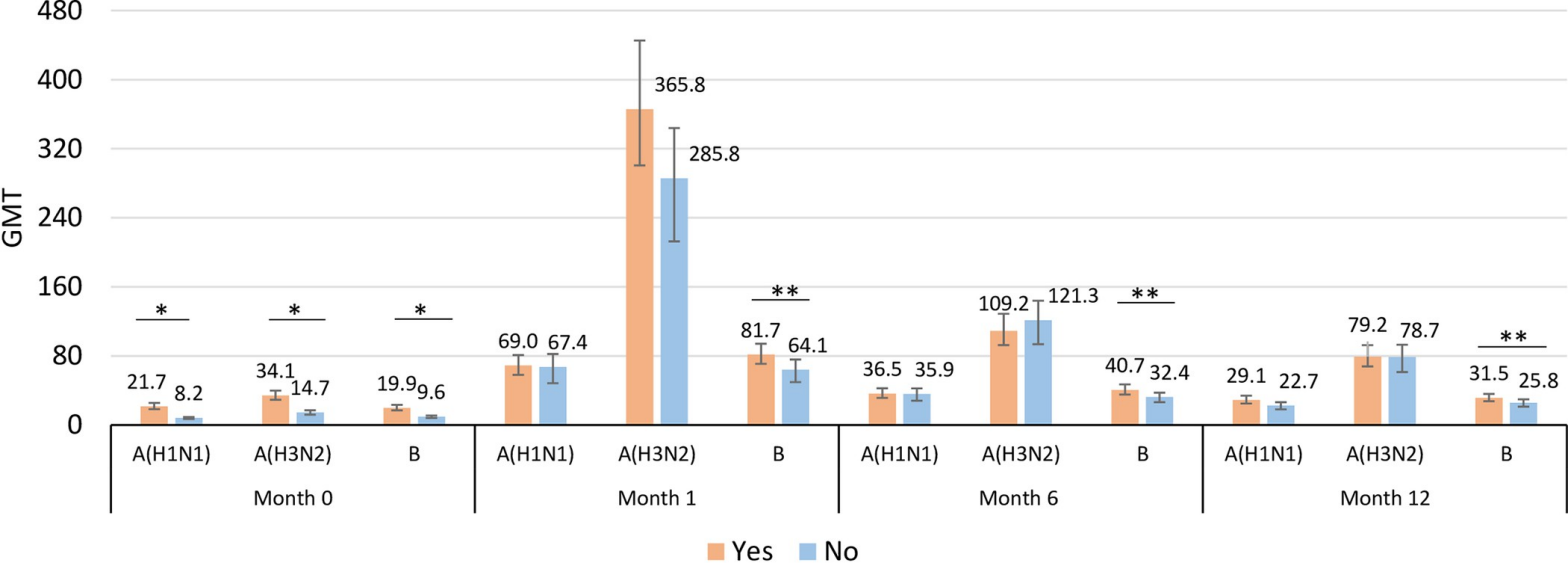
Geometric Meam Toter (GMT) among previously vaccinated group (Yes) and previously unvaccinated group (No) of IIV3 in 2014–2015 seson at study baseline 1-month, 6-month and 12-month follow-up. * Significance level p-value <0.001, ** Significance level p-value <0.05 from generalized linear model with identity function on logged transformed titer adjusted for age group and sex.

Baseline GMTs of previously vaccinated participants (n = 203) were significantly higher than those who had not been vaccinated in 2014 (n = 167) against A(H1N1)pdm09 (21.7 vs 8.2), A(H3N2) (34.1 vs 14.7), and B (19.9 vs 9.6) (all p<0.001) (Fig 2). Post-vaccination GMTs were similar in both groups for A(H1N1) and A(H3N2) at all the time points (all p-values >0.05).

In case of influenza B, the previously vaccinated group had significantly higher GMT than the previously unvaccinated group at all three time points (all p<0.05) (Fig 2).

#### b) Seroprotection

At baseline, the previously vaccinated group had a higher proportion of participants with seroprotective antibody levels than the previously unvaccinated group against A(H1N1)pdm09 (40.4% vs 12.0%, adjusted RR 3.38, 95% CI 2.17–5.30), A(H3N2) (54.2% vs 24.6%, aRR 2.33, 95% CI 1.74–3.12), and B (37.0% vs 15.6%, aRR 2.50, 95% CI 1.69–3.70) (Table 3). After one month, seroprotection rates increased and were similar in both groups for A(H1N1)pdm09 (78.2% vs 69.9%, aRR 1.10, 95% CI 0.97–1.25), and A(H3N2) (97.0% vs 90.4%, aRR 1.08, 95% CI 0.87–1.33) but higher in those previously vaccinated for B (87.1% vs 74.1%, aRR 1.20, 95% CI 1.08–1.32). At six months, the proportion of participants with seroprotection was only different against influenza B (64.7% in the previously vaccinated group vs 54.6% in the previously unvaccinated group, aRR 1.21, 95% CI 1.02–1.43). Both groups had less than 60% seroprotection against A(H1N1)pdm09 at 6 months; for A(H3N2), ≥78% seroprotection was maintained for 12 months in both groups (Table 4).

**Table 4:** Prevalence of sero-protection in elderly vaccinees 65 years and older who previously vaccinated group (Yes) or previously unvaccinated group (No) of IIV3 in 2014–2015 season.

Vaccine strain	Sero-protection by previously vaccinated TIV in 2014–2015 season							
	Month 0 (n = 370)		Month 1 (n = 368)		Month 6 (n = 363)		Month 12 (n = 360)	
	Yes (n = 203)	No (n = 167)	Yes (n = 202)	No (n = 166)	Yes (n = 198)	No (n = 165)	Yes (n = 196)	No (n = 164)
A(H1N1) n (%)	80 (40.4)	20 (12.0)	158 (78.2)	116 (69.9)	116 (58.6)	90 (54.6)	98 (50.0)	71 (43.3)
Adjusted RR (95% CI)	3.38 (2.17, 5.30)		1.10 (0.97, 1.25)		1.06 (0.88, 1.27)		1.13 (0.90, 1.42)	
p-value^α^	< 0.001		0.123		0.569		0.292	
A(H3N2) n (%)	110 (54.2)	41 (24.6)	196 (97.0)	150 (90.4)	172 (86.9)	139 (84.2)	159 (81.1)	128 (78.1)
Adjusted RR (95% CI)	2.33 (1.74, 3.12)		1.08 (0.87, 1.33)		1.03 (0.95, 1.13)		1.03 (0.93, 1.15)	
p-value^α^	< 0.001		0.507		0.483		0.575	
B n (%)	75 (37.0)	26 (15.6)	176 (87.1)	123 (74.1)	128 (64.7)	90 (54.6)	104 (53.1)	74 (45.1)
Adjusted RR (95% CI)	2.50 (1.69, 3.70)		1.20 (1.08, 1.32)		1.21 (1.02, 1.43)		1.22 (0.99, 1.51)	
p-value^α^	< 0.001		< 0.001		0.028		0.064	

RR = relative risk. CI = confidence interval

^α^Log-binomial regression adjusted for age group and sex. Significance level at < 0.05.

#### c) Seroconversion

**Fig 3:**
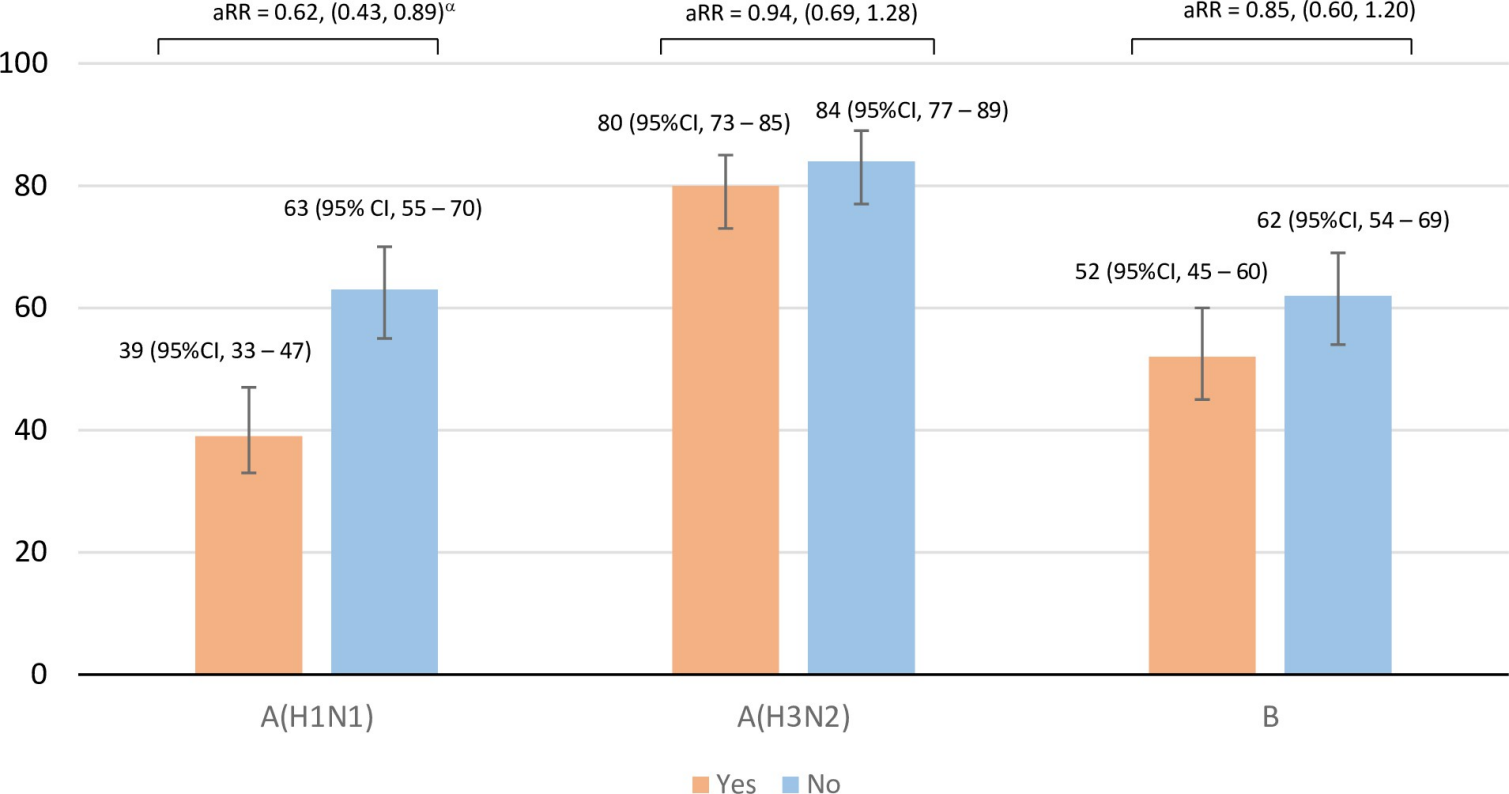
Prevalence of sero-conversion at 1 month among previously vaccinated group (Yes) and previously unvaccinated group (No) of IIV3 in 2014–2015 season. aRR = adjusted relative risk, Log-binomial regression adjusted for age group and sex, CI = confidence interval, ^α^Significance level at <0.05.

Seroconversion was lower in the previously vaccinated group than the previously unvaccinated group for A(H1N1)pdm09 at one month post-vaccination (39% vs 63%, aRR 0.62, 95% CI 0.43–0.89) (Fig 3). This was due to the high proportion of vaccinated participants who had already antibodies at month 0. The seroconversion rates were not different among the two groups for A(H3N2) (80% vs 84%, aRR 0.94, 95% CI 0.69–1.28) and B (52% vs 62%, aRR 0.85, 95% CI 0.60–1.20) (Fig 3).

## Discussion

We measured the immunogenicity of IIV3 using HI assay among a cohort of community-dwelling Thai older adults aged ≥65 years during the 2015–16 influenza season and found that IIV3 elicited a good humoral response. The antibody response was not blunted in persons with prior vaccination as a higher proportion of previously vaccinated participants had HI titers ≥1:40 against B after one and six months of vaccination. Nonetheless, seroconversion was significantly lower in persons previously vaccinated for A(H1N1)pdm09 in 2014, but this was not observed for influenza A(H3N2) and influenza B in which H1N1 is an ‘old’ antigen, used the vaccine the year before, whereas H3N2 and B are in part ‘new’ antigens, as the vaccine strains have changed. Previous vaccination in 2014 was not associated with any diminished seroprotetion, with 50% or more having HI titers ≥1:40 at 12 months with no difference between vaccinated or unvaccinated. These findings provide important empirical support for the national policy of annual recommendation of IIV3 for older adults aged ≥65 years in Thailand.

In our study, immune responses against all three influenza strains were strong after one month of vaccination, exceeding the Committee for Proprietary Medicinal Products (CPMP) recommended serological criteria for influenza vaccine for adults aged over 60 years (i.e. >30% seroconversion rate, >60% seroprotection rate). Our findings differ from a previous Thai study among older adults in which the seroprotection rates against influenza B did not meet the CPMP criteria [27]. While a higher percentage of persons maintained seroprotective HI titers against influenza A(H3N2) throughout the study period, seroprotection against influenza A(H1N1)pdm09 and B declined below the CPMP level within six months of vaccination. Nonetheless, we did not find an association of this decline with advancing age. While intra-seasonal waning of influenza vaccine-induced protection has been reported consistently in the published literature, it does not seem to be restricted only to older adults [13,31,32]. Our findings add to the studies that suggest that intra-seasonal decline in humoral immune responses may not be strictly related to age [13].

Although prior vaccination with IIV3 has been linked with lower humoral immune response in some studies due to higher pre-existing serum antibodies, the clinical effect of prior vaccination is still controversial [33]. A higher baseline antibody titer may have resulted in a higher proportion of previously vaccinated participants reaching seroprotective HI titers after the new vaccination in our study. The weaker seroconversion for influenza A(H1N1)pdm09 in previously vaccinated participants may be due to the small antigenic distance as the vaccine strain for A(H1N1)pdm09 remained unchanged in 2014 and 2015 [26]. The antigenic distance hypothesis (ADH) posits that prior vaccination can cause negative interference on the protection provided by the current season’s vaccine when the antigenic distance is small between the prior and current vaccines but large between the prior season vaccine and the currently circulating strains, effect of repeated influenza vaccination were consistent with the ADH and may contributed to findings of low relative vaccine effectiveness [34], which we had been observed significance lower seroconversion for unchanged vaccine strain A(H1N1)pdm09 in previously influenza vaccinated group. It has been posited that repeated vaccination with the same vaccine strains from year to year may reduce the production of antibody-secreting cells and memory B cells [35]. Whereas for influenza A(H3N2) and B, the vaccine strains were different between 2014 and 2015, and this might have induced a robust B cell response and produced better immune responses in previously vaccinated participants than for influenza A(H1N1)pdm09.

There are few limitations in our study. First, we did not include other variables that may affect the immunological response in older adults such as chronic medication [36], obesity, and other age-related changes. However, frailty, which is often a confounding factor in vaccine effectiveness studies among older adults, has been ruled out as a factor that reduces influenza vaccine-induced antibody responses among community-dwelling older adults [37]. Second, we did not assess the effect of number of prior vaccinations on the immune response. It is likely that the older Thai adults in our sample may not have received many vaccines before 2014 which might have affected our results. However, we could not verify the participant-reported prior vaccination status before 2014 with the NHSO database, therefore did not use them in our analysis. Third, we could not include younger adults in the cohort to accurately compare the effect of age on the immune responses as they are not considered a high-risk group eligible for the free seasonal influenza vaccines provided by the government in Thailand. Finally, we did not assess cell-mediated immune responses which may correlate better with vaccine-induced protection in older adults than HI titers [38].

## Conclusion

In conclusion, seasonal trivalent inactivated influenza vaccination elicited a good humoral response in Thai older adults with a longer duration of seroprotection against A(H3N2) than the other two strains. While seroconversion may have been attenuated in persons previously vaccinated for influenza A(H1N1)pdm09, this was not apparent for influenza A(H3N2) and influenza B; and prior vaccination was not associated with any diminished seroprotection. Further studies are needed to explore the long-term effect of influenza vaccination among older Thai adults.

## Supporting information

S1 Checklist (DOCX)

S1 File (XLS)

S2 File (PDF)
