# Supplementary material for: Does prior vaccination affect the immune response to seasonal influenza vaccination among older adults? Findings from a prospective cohort study in a Northeastern Province of Thailand
Source: PLoS One. 2023 Feb 3;18(2):e0279962. doi: 10.1371/journal.pone.0279962 (PMC9897550; doi:10.1371/journal.pone.0279962)
Supplement: S1 Checklist — (DOCX) [file pone.0279962.s001.docx]

| **TREND Statemen Checklist** | | | | |
| --- | --- | --- | --- | --- |
| **Paper Section/Topic** | **Item No.** | **Descriptor** | **Reported?** | |
|  |  |  | **√** | **Pg#** |
| **Title and Abstract** | | | | |
| Title and Abstract | 1 | Information on how unit were allocated to interventions | Title “Does prior vaccination affect the immune response to seasonal influenza vaccination among older adults? Findings from a prospective cohort study in a Northeastern Province of Thailand” | 1 |
|  |  | Structured abstract recommended | Yes | 1 |
|  |  | Information on target population or study sample | Older adult in Nakhon Phanom province, Thailand |  |
| **Introduction** | | | | |
| Background | 2 | Scientific background and explanation of rationale | Older adults are at a disproportionately higher risk of influenza-associated morbidity and mortality than their younger counterparts. In older adults, age-related changes can gradually deteriorate the capacity to develop vaccine-induced immunity through a process known as immunosenescence. However, the results of immunogenicity studies, which test the antibody response of influenza vaccines by quantifying the amount of antibody seroconversion and duration of humoral immune response after influenza vaccination, have not been consistent among older adults | 3 |
|  |  | Theories used in designing behavioral interventions | There is no theory in this study. Based on main study to evaluate vaccine effectiveness it needed vaccine coverage in 50% among this study group in community. Thus, we had a campaign to increase coverage from approximately 305 up to 50%. We published our result in PLOS ONE available at  [pone_0188422 1..13 (plos.org)](https://journals.plos.org/plosone/article/file?id=10.1371/journal.pone.0188422&type=printable) | - |
| **Methods** | | | | |
| Participants | 3 | Eligibility criteria for participants, including criteria at different levels in recruitment/sampling plan (e.g., cities, clinics, subjects) | This prospective longitudinal study was nested in a larger cohort study that measured the effectiveness of the trivalent inactivated influenza vaccine in community-dwelling persons aged 65 years and older in Nakhon Phanom, Thailand.  At the district level, it was chosen by vaccine coverage criteria in 40-50%.  At the individual, inclusion criteria include:  The participants had to be residing in the community for at least a year and able to communicate with the study staff to be included in the study. Institutionalized persons, prior recipients of the vaccine during 2015 (but before the study), those with an acute medical condition, a history of a bleeding disorder, a known allergy to influenza vaccine or egg, or any contraindication to venipuncture were excluded from the study | 5 |
|  |  | Method of recruitment (e.g., referral, self-selection), including the sampling method if a systematic sampling plan was implemented | Participants who visited sub-district health centers and district hospitals requesting vaccination against influenza in 2015 were enrolled in this study using a convenience sampling method | 5 |
|  |  | Recruitment setting | sub-district health centers and district hospitals | 5 |
|  |  | Settings and locations where the data were collected | sub-district health centers and district hospitals | 5 |
| Interventions | 4 | Details of the interventions intended for each study condition and how and when they were actually administered, specifically including: | In this study there was no intervention but for measuring the antibody response following the objective the participant had to receive influenza vaccine. | - |
|  |  | o Content: what was given? | Influenza vaccination was provided free of charge via a national campaign at sub-district health centers and district hospitals in May 2015.   - Nurse at at sub-district health centers and district hospitals were vaccinators - Vaccination was done in May 2015 that was before influenza season starting. | 5 |
|  |  | o Delivery method: how was the content given? |  |  |
|  |  | o Unit of delivery: how were the subjects grouped during delivery? |  |  |
|  |  | o Deliverer: who delivered the intervention? |  |  |
|  |  | o Setting: where was the intervention delivered? |  |  |
|  |  | o Exposure quantity and duration: how many sessions or episodes or events were intended to be delivered? How long were they intended to last? | Vaccination only one dose and follow up blood draw one year. | - |
|  |  | o Time span: how long was it intended to take to deliver the intervention to each unit? | N/A |  |
|  |  | o Activities to increase compliance or adherence (e.g., incentives) | Blood samples were obtained by research nurses at the participant's home | 6 |
| Objectives | 5 | Specific objectives and hypotheses | We measured the immunogenicity of seasonal trivalent inactivated influenza vaccines (IIV3) among older Thai adults and the effect of one-year prior vaccination status on immune responses. | 2 |
| Outcomes | 6 | Clearly defined primary and secondary outcome measures | Serological outcomes  Seroprotection was defined as HI titers of 1:40 or greater; whereas everyone with a baseline HI titer ≥1:10 who had a four-fold increase in HI antibody count post-vaccination, or who had a baseline HI titer <1:10 and had a post-vaccination count ≥1:40 were considered seroconverted against influenza. The geometric mean titer (GMT) was calculated by taking the antilog of the mean of logarithmically transformed HI titers. Geometric mean ratio (GMR) was calculated as the ratio of GMT of post-vaccination blood /GMT of pre-vaccination blood | 6 |
|  |  |  |  |  |
|  |  | Methods used to collect data and any methods used to enhance the quality of measurements | Enrollment questionnaires were administered by trained health volunteers and study team members at the sub-district health centers and district hospitals. Vaccination in 2014 was verified with the National Health Security Office (NHSO) database. The participants were vaccinated with IIV3 in the 2015-16 season and blood samples (5ml) were collected from the participants at four intervals during the study period by the study nurses. The first blood sample was collected prior to the vaccination, while the 2^nd^, 3^rd^, and 4^th^ samples were collected at 1, 6, and 12 months thereafter. Blood samples were obtained by research nurses at the participant's home and transported back to the district hospital in ice packs for those who were unable to travel to the health facility. At the district hospital, sera were separated from the whole blood samples and stored at -20° C until tested. The serum samples were sent every week to a laboratory at the Thai National Institutes of Health (NIH). | 5-6 |
|  |  | Information on validated instruments such as psychometric and biometric properties | N/A |  |
| Sample Size | 7 | How sample size was determined and, when applicable, explanation of any interim analyses and stopping rules | A sample size of 384 was calculated in OpenEpi (www.OpenEpi.com) assuming a 50% seroconversion rate, the estimated size of the population of adults aged ≥65 years of the two districts to be 15,000, 5% type I error, and absolute precision of 5 percentage points. | 7 |
| Assignment Method | 8 | Unit of assignment (the unit being assigned to study condition, e.g., individual, group, community) | Group | - |
|  |  | Method used to assign units to study conditions, including details of any restriction (e.g., blocking, stratification, minimization) | Everyone received vaccine by its voluntary following the national campaign | - |
|  |  | Inclusion of aspects employed to help minimize potential bias induced due to non-randomization (e.g., matching) | Individual who participated in this study would be a person who was more likely to access vaccination easier than who did not. However, we conducted a population-based, cross-sectional survey to measure vaccine coverage and identify factors associated with influenza vaccination among older Thai adults that could bias measures of vaccine effectiveness. The strongest predictors of vaccination were distance to the nearest vaccination center (PR 3.0, 95% CI 1.7–5.1 for participants in the closest quartile compared to the furthest), and high levels of a perception of benefits of influenza vaccination (PR 2.8, 95% CI 1.4–5.6) and cues to action (PR 2.7, 95% CI 1.5–5.1). Publication available at  [pone_0188422 1..13 (plos.org)](https://journals.plos.org/plosone/article/file?id=10.1371/journal.pone.0188422&type=printable)  Nakhon Phanom provincial health office used this result such as “distance to the nearest vaccination center” changing the policy of vaccination campaign. Normally the vaccination will take place in only district hospital but for this project the vaccination can be in sub district health facility. |  |
| Blinding (masking) | 9 | Whether or not participants, those administering the interventions, and those assessing the outcomes were blinded to study condition assignment; if so, statement regarding how the blinding was accomplished and how it was assessed. | N/A |  |
| Unit of Analysis | 10 | Description of the smallest unit that is being analyzed to assess intervention effects (e.g., individual, group, or community) | Group | - |
|  |  | If the unit of analysis differs from the unit of assignment, the analytical method used to account for this (e.g., adjusting the standard error estimates by the design effect or using multilevel analysis) | N/A |  |
| Statistical Methods | 11 | Statistical methods used to compare study groups for primary methods outcome(s), including complex methods of correlated data | The 95% confidence intervals for GMTs were calculated from the Student’s t distribution of log10-transformed titers.    We determined the association between 2014 vaccination and GMTs and GMRs at each time point using generalized linear regression on logged titers, adjusting for age and sex.    Estimates of seroprotection and seroconversion by vaccination history were also adjusted for age and sex using logistic regression. To determine the association of age on the duration of the immune response, we dichotomized age (<75 years/≥75 years) and assessed the difference in seroprotection rate at each time point, adjusting for sex and prior vaccination  The significance was set at a p-value <0.05. | 7 |
|  |  | Statistical methods used for additional analyses, such as a subgroup analyses and adjusted analysis |  |  |
|  |  | Methods for imputing missing data, if used | N/A |  |
|  |  | Statistical software or programs used | STATA software version 14.2 (StataCorp LP, College Station, TX, USA) | 7 |
| **Results** | | | | |
| Participant flow | 12 | Flow of participants through each stage of the study: enrollment, assignment, allocation, and intervention exposure, follow-up, analysis (a diagram is strongly recommended) | Figure 1 | T. 6 |
|  |  | o Enrollment: the numbers of participants screened for eligibility, found to be eligible or not eligible, declined to be enrolled, and enrolled in the study | Enrolled 384  Excluded from analysis 14; because no have vaccination history  Total in analysis was 370 | 7 |
|  |  | o Assignment: the numbers of participants assigned to a study condition | Divided 370 participants into 2 group;   - 203 had prior vaccination in 2014 - 167 participants had no prior vaccination in 2014 | 7 |
|  |  | o Allocation and intervention exposure: the number of participants assigned to each study condition and the number of participants who received each intervention |  |  |
|  |  | o Follow-up: the number of participants who completed the followup or did not complete the follow-up (i.e., lost to follow-up), by study condition | Among 203 participant who had prior vaccination history in 2014, 203, 202, 198 and 196 participants completed blood draw at baseline, one month, 6 months and 12 months after vaccination respectively.  Among 167 participant who had no prior vaccination history in 2014, 167, 166, 165 and 164 participants completed blood draw at baseline, one month, 6 months and 12 months after vaccination respectively. | T.6 |
|  |  | o Analysis: the number of participants included in or excluded from the main analysis, by study condition | By intention to treat we kept the number of participants of each time point of blood draw in the analysis | - |
|  |  | Description of protocol deviations from study as planned, along with reasons | Fig 1 showed the number of participants who completed activity at each time point.  Of 384 participants, we excluded 3 participants who got influenza infection during a follow up period and 11 participant who had no vaccination record. | T.6 |
| Recruitment | 13 | Dates defining the periods of recruitment and follow-up | Vaccination was provided in May, 2015.  Participants were followed up for 12 months in the 2015-16 influenza season (from May 2015 through May 2016) | 5 |
| Baseline Data | 14 | Baseline demographic and clinical characteristics of participants in each study condition | Due to this longitudinal study was nested in a larger cohort study, we did not present the baseline demographic in the table but described some characteristics such as ages distribution, sex, vaccination history. | 8 |
|  |  | Baseline characteristics for each study condition relevant to specific disease prevention research | We compared vaccination history by age and sex. | 8 |
|  |  | Baseline comparisons of those lost to follow-up and those retained, overall and by study condition | N/A |  |
|  |  | Comparison between study population at baseline and target population of interest | N/A |  |
| Baseline equivalence | 15 | Data on study group equivalence at baseline and statistical methods used to control for baseline differences | For GMTs and GMRs at each time point using generalized linear regression on logged titers, adjusting for age and sex.  Using logistic regression to estimates of seroprotection and seroconversion by vaccination history were also adjusted for age and sex and to determine the association of age on the duration of the immune response, we dichotomized age (<75 years/≥75 years) and assessed the difference in seroprotection rate at each time point, adjusting for sex and prior vaccination.  Results were presented in fig 3,4 and table table 2 | 8-9  T.2, 8 |
| Numbers analyzed | 16 | Number of participants (denominator) included in each analysis for each study condition, particularly when the denominators change for different outcomes; statement of the results in absolute numbers when feasible | -203 participants had prior vaccination in 2014  -167 participants had no prior vaccination in 2014 |  |
|  |  | Indication of whether the analysis strategy was “intention to treat” or, if not, description of how non-compliers were treated in the analyses | Yes | 7 |
| Outcomes and estimation | 17 | For each primary and secondary outcome, a summary of results for each estimation study condition, and the estimated effect size and a confidence interval to indicate the precision | N/A |  |
|  |  | Inclusion of null and negative findings |  |  |
|  |  | Inclusion of results from testing pre-specified causal pathways through which the intervention was intended to operate, if any |  |  |
| Ancillary  analyses | 18 | Summary of other analyses performed, including subgroup or restricted  analyses, indicating which are pre-specified or exploratory | Compared seroprotective levels between participants aged <75 years and aged ≥75 years | 9 |
| Adverse events | 19 | Summary of all-important adverse events or unintended effects in each  study condition (including summary measures, effect size estimates, and  confidence intervals) | N/A |  |
| **DISCUSSION** | | | | |
| Interpretation | 20 | Interpretation of the results, taking into account study hypotheses, sources of potential bias, imprecision of measures, multiplicative analyses, and other limitations or weaknesses of the study | We measured the immunogenicity of IIV3 using HI assay among a cohort of community-dwelling Thai older adults aged ≥65 years during the 2015-16 influenza season and found that IIV3 elicited a good humoral response. While previously unvaccinated persons had higher GMR throughout the study. Previous vaccination in 2014 was not associated with any diminished seroprotection, with 50% or more having HI titers ≥1:40 at 12 months with no difference between vaccinated or unvaccinated  seroconversion was significantly lower in persons previously vaccinated for A(H1N1)pdm09 in 2014, but this was not observed for influenza A(H3N2) and influenza B.  There are few limitations in our study.  First, we did not include other variables that may affect the immunological response in older adults such as chronic medication, obesity, and other age-related changes. However, frailty, which is often a confounding factor in vaccine effectiveness studies among older adults, has been ruled out as a factor that reduces influenza vaccine-induced antibody responses among community-dwelling older adults.  Second, we did not assess the effect of number of prior vaccinations on the immune response. It is likely that the older Thai adults in our sample may not have received many vaccines before 2014 which might have affected our results. However, we could not verify the participant-reported prior vaccination status before 2014 with the NHSO database, therefore did not use them in our analysis.  Third, we could not include younger adults in the cohort to accurately compare the effect of age on the immune responses as they are not considered a high-risk group eligible for the free seasonal influenza vaccines provided by the government in Thailand.  Finally, we did not assess cell-mediated immune responses which may correlate better with vaccine-induced protection in older adults than HI titers. | 11  13 |
|  |  | Discussion of results taking into account the mechanism by which the intervention was intended to work (causal pathways) or alternative mechanisms or explanations | In our study, immune responses against all three influenza strains were strong after one month of vaccination, exceeding the Committee for Proprietary Medicinal Products (CPMP) recommended serological criteria for influenza vaccine for adults aged over 60 years (i.e. >30% seroconversion rate, >60% seroprotection rate). | 12 |
|  |  | Discussion of the success of and barriers to implementing the intervention, fidelity of implementation | Our findings differ from a previous Thai study among older adults in which the seroprotection rates against influenza B did not meet the CPMP criteria. | 12 |
|  |  | Discussion of research, programmatic, or policy implications | These findings provide important empirical support for the national policy of annual recommendation of IIV3 for older adults aged ≥65 years in Thailand. | 11 |
| Generalizability | 21 | Generalizability (external validity) of the trial findings, taking into account the study population, the characteristics of the intervention, length of follow-up, incentives, compliance rates, specific sites/settings involved in the study, and other contextual issues | This study followed up the participant 1 year that covered influenza seasoning in Thailand. | - |
| Overall Evidence | 22 | General interpretation of the results in the context of current evidence and current theory | In conclusion, seasonal trivalent inactivated influenza vaccination elicited a good humoral response in Thai older adults with a longer duration of seroprotection against A(H3N2) than the other two strains. While seroconversion may have been attenuated in persons previously vaccinated for influenza A(H1N1)pdm09, this was not apparent for influenza A(H3N2) and influenza B; and prior vaccination was not associated with any diminished seroprotection.  Further studies are needed to explore the long-term effect of influenza vaccination among older Thai adults | 13 |
