# Supplementary material for: Does prior vaccination affect the immune response to seasonal influenza vaccination among older adults? Findings from a prospective cohort study in a Northeastern Province of Thailand
Source: PLoS One. 2023 Feb 3;18(2):e0279962. doi: 10.1371/journal.pone.0279962 (PMC9897550; doi:10.1371/journal.pone.0279962)
Supplement: S2 File — (PDF) [file pone.0279962.s003.pdf]

1. Title of the Project

Measuring the effectiveness of the trivalent inactivated influenza vaccine in persons aged 65 years and older in Nakhon Phanom, Thailand

2. Name and address of Principal investigator(s), co-investigator(s) and advisor(s)

Principal Investigator

Dr. Kriengkrai Prasert MD

Nakhon Phanom Public Health Office

Aphibanbuncha Road, Muang District

Nakhon Phanom, 48000

Telephone:042511410   Office: 081- 975-5460

Co-Investigators

Thammasat University

Prof. Dr. Jayanton Patumanond MD MPH MSc PhD

Thammasat University

Clinical Research Center

4th floor, Rajsuda building

Pathum Thani 12120

Telephone: Office: 02-564-4444 ext 7536

Thailand Ministry of Public Health

Health Technical Office

Dr. Kamjad Ramakul MD

Dr. Supakit Sirirak MD MPHM

Surut Chatchaiyalerk MBA

Thailand Ministry of Public Health (MOPH)

PS Building

Tivanon Rd., Nonthaburi 11000, Thailand

Telephone: Office: 02-590-1717

National Institute of Health (NIH)

Dr. Somchai Sangkitporn MD

Malinee Chittaganpich MSc

Sunthareeya Waicharoen MS

Thailand Ministry of Public Health (MOPH)

NIH Building

Tivanon Rd., Nonthaburi 11000, Thailand

Telephone: Office: 02-589-9850

Nakhon Phanom Public Health Office (PHO)

Dr. Prapas Weerapol MD

Dr. Preeda Worahan MD

Paiwan Laowattanathaworn MPH

Dr. Sirirak Jaichuang PhD

Aphibanbuncha Road, Muang District

Nakhon Phanom, 48000

Telephone: Office: 042-511410

Nakhon Phanom Provincial Hospital

Dr. Yuthachai Yrisakul MD

Dr. Supachok Chemla MD

Chuwattana Chara BSN

Aphibanbuncha Road, Muang District

Nakhon Phanom, 48000

Telephone: Office: 042-511424

Thailand MOPH – U.S. CDC Collaboration (TUC)

Dr. Kim Lindblade PhD MPH

Dr. Prabda Prapasiri DrPH

Darunee Ditsungnoen MPH

Dr. Sonal Goyal PharmD MPH

Thailand MOPH-US CDC Collaboration

DDC Building 7 MOPH Tivanon Rd., Nonthaburi 11000, Thailand

Telephone: Office: 02-580-0669

US Centers for Disease Control and Prevention (CDC)

Dr. Fatimah Dawood MD

Centers for Disease Control and Prevention

1600 Clifton Road, Building 24

Atlanta, GA 30329-4027 USA

3. Project Summary

3.1 Brief Summary

3.1.1 Introduction, background, objectives

Thailand targets high risk groups for annual immunization with trivalent inactivated influenza vaccine (IIV3), including persons aged 65 years and older. However, there are few data from Thailand on the effectiveness of IIV3 in preventing influenza-associated illness and hospitalizations, and even fewer data on the cost-effectiveness of vaccination. This is a prospective population-based cohort study of persons aged 65 years and older that will compare the incidence of influenza-associated acute respiratory infection (ARI) and influenza-associated severe acute respiratory infection (SARI) between individuals who do and do not receive the influenza vaccine to measure the reduction in disease associated with IIV3. We will enroll and collect nasal self-swabs from randomly selected elderly each time they experience an ARI in two districts of Nakhon Phanom and test them for influenza. If they are hospitalized with SARI, a nurse will take a nasopharyngeal swab to test for influenza. Data on factors associated with vaccination will be measured and propensity for vaccination will be modeled in order to account for potential biases in the analysis. We will follow these elderly participants for two years and compare the number of influenza-associated ARI and SARI events between elderly that were vaccinated and elderly that were not vaccinated to calculate how effective IIV3 was in preventing influenza. This study will also evaluate the cost-effectiveness of IIV3 vaccine. Additionally, this study will measure the effect of having ARIs/SARIs on functional status, the VE of IIV3 in preventing influenza associated exacerbations among participants with chronic obstructive pulmonary disease(COPD), the duration of adequate IIV3 antibody levels in blood, and the incidence of respiratory syncytial virus (RSV) among the elderly.

3.1.2 Location of the study

The study will be conducted in Nakhon Phanom (NP) Province. This province was selected because of a history of collaborating with TUC on research and surveillance and an interest from the Provincial Health Office to increase influenza vaccination coverage among the elderly. Nakhon Phanom has 12 districts, 10 of which are suitable for studies (two districts had a high probability of becoming flooded during the rainy season making movements difficult). In 2014, 4 of the 10 districts were systematically selected to be involved in the Influenza Vaccination Campaign run by the MOPH where high-risk community members were encouraged to become vaccinated; a similar campaign is being planned for 2015. Our study will sample from two of the four districts involved in the campaign to ensure that there are enough elderly members who became vaccinated in these districts to measure vaccine effectiveness. The two districts are That Phanom and Plapak which were chosen due to feasibility of location and consent of district health directors.

3.1.3 Duration of the study

We will begin recruiting and training healthcare volunteers, nurse practitioners and nurses on data collection and laboratory technique in February of 2015 through April 2015. Participants will be enrolled in the study by June 2015 and followed for two years. Additionally, approximately six more months will be needed after the study to analyze data, which will begin in June 2017. The entire study will take approximately 2 years and 10 months.

3.1.4 Study design, methodology, study plan

Persons aged 65 years and older will be recruited in the two selected districts of Nakhon Phanom Province and followed weekly for one year to identify any new episodes of ARI or SARI. Baseline information will be collected from all participants, including functional status and frailty indicators, chronic diseases, smoking history, and variables related to socioeconomic status. Participants will be asked how far the closest health center is from them and household latitude and longitude will be collected in order to calculate distances to medical facilities. ARI will be defined as a new onset of cough or worsening of chronic cough, with or without fever.

SARI will be defined as new onset of cough, worsening of chronic cough or difficulty breathing with a fever ≥ 38^0 Celsius that requires hospitalization. Nasal and nasopharyngeal samples taken from persons with ARI or SARI will be tested using real-time reverse-transcription polymerase chain reaction (rRT-PCR) for influenza and respiratory syncytial viruses. The incidence of laboratory-confirmed, influenza-associated ARI and SARI will be compared between vaccinated and unvaccinated cohort participants, controlling for baseline functional status and underlying medical conditions that might confound the primary association. Data on costs of visits for ARI and SARI for pneumonia or acute respiratory illness will be collected, along with information on the costs of the vaccination campaign, to determine the cost per influenza episode averted and the value for money of the immunization campaign. Results will assist the Thai Ministry of Public Health to make informed decisions related to improving vaccination coverage among the elderly in Thailand.

In addition to vaccine effectiveness and cost effectiveness data, this study will also take the opportunity to measure the effect of ARI and SARI on functional status among the elderly, measure the incidence of influenza-associated COPD exacerbations among those with ARI or SARI, evaluate humoral immune response of IIV3 through measurement of hemagglutination inhibition assay (HAI), and measure the incidence of respiratory syncytial virus (RSV) among the elderly with ARI or SARI.

Vaccination: Cohort participants, along with all persons aged 65 years and older in the districts identified by the MOPH for increased IIV3 coverage, will be eligible for influenza vaccination through routine vaccine delivery strategies used by the MOPH. No vaccine will be provided as part of this protocol. The study will be enrolling participants from two districts in Nakhon Phanom, That Phanom and Plapak. According to the Nakhon Phanom Provincial Health Office, the vaccination rate in 2014 was calculated to be 52% and 28% in That Phanom and Plapak, respectively. This VE study will follow a pilot program of the MOPH to increase vaccination among the elderly through a concentrated and focused vaccine campaign in the elderly. This year, due to planned vaccination campaigns, we expect the vaccination rate to be around 50% in both districts. A companion study to this protocol, already underway, will evaluate the coverage achieved by the pilot campaign as well as measure factors associated with receipt of the vaccine in this age group.

Influenza surveillance: Contact information, including phone numbers, will be recorded for all cohort participants. Health volunteers will contact all participants weekly for one year to identify any individual with new onset of cough or worsening of chronic cough, with or without fever (ARI) and any individuals with cough and fever≥38^0C requiring hospitalization (SARI). There are a number of diagnostic specimens that have been used to identify the viral etiology of ARI/SARI, including nasal, nasopharyngeal (NP) and oropharyngeal (OP) swabs. The most acceptable of these to patients is the nasal swab, as it is less invasive (1). Individuals with new onset of cough or worsening of chronic cough will be asked to take a nasal self-swab and properly store the sample in a separate tube. If the participant is too ill to be able to take a self-nasal swab, they will ask their assigned health volunteer to schedule a time to assist them in taking the swab. They will also be encouraged to seek medical attention at the sub-district health center, district hospital or provincial hospital where trained study staff is located if they are feeling very ill. The study will facilitate transport to the local health center for any severely ill participants who are unable to transport themselves. Trained research nurses in the hospital will take nasopharyngeal swabs for influenza diagnosis from admitted participants, and routine clinical information will be recorded. Nasopharyngeal swabs have been shown to be comparable to oropharyngeal swabs for detecting Influenza A and equal or superior to oropharyngeal swabs for detecting Influenza B (2, 3).

Laboratory testing of nasal and nasopharyngeal swabs: Nasal self-swabs from participants will be stored in transport media (TM) and immediately transported with ice packs to the sub-district health center by a health volunteer and placed into a refrigerator monitored to be between 2 to 8^0C for a maximum of 24 hours. From there, the specimens will be transported to the district hospital in ice packs where they will be stored in liquid nitrogen tanks within 24 hours. Nasopharyngeal swabs taken by nurses will also be stored at the local health center in a regulated refrigerator for a maximum of 24 hours and transported to the district hospital with ice packs to be stored in liquid nitrogen tanks. All respiratory specimens will then be transported to the MOPH National Institute of Health (NIH) laboratory in Nonthaburi in liquid nitrogen tanks where they will be tested using rRT-PCR for influenza and RSV using protocols developed by CDC (4). If specimens will not be tested immediately upon arrival at the laboratory, they will be stored below -70^0 C until tested.

Laboratory testing of blood samples: In order to measure hemagglutination inhibition assay (HAI) antibody titers to check for duration of seroconversion in elderly from IIV3, blood samples will be requested from elderly arriving to the district hospitals vaccination clinics in That Phanom and Plapak for vaccination. The first sample will be taken just before vaccine is administered on day 0 (as a baseline measurement) and on day 28 (after 1 month), day 180 (after 6 months) and day 360 (after 1 year). One additional blood draw will be requested after 28 days of vaccination in the second year to compare the effect of prior vaccination on level of immunological response. Participants will be expected to go to their district hospital to have their scheduled blood draw. Blood will be drawn at the participant's home if they are unable to travel to their district hospital by a research nurse. Blood samples will be collected and immediately sent to the district hospital lab to separate the serum from the blood (if blood is collected at a participant's home, it can be transported back to the district hospital with the nurse at room temperature). The serum will be stored in a refrigerator between 4-8^{0}C for no longer than seven days and transported to NIH on a weekly basis. During transport to NIH, serum samples will be stored with ice packs for less than 24 hours. Upon reaching NIH, the samples will be measured using a standard HAI (5). Seroconversion will be measured as the percent of participants who have one of the following: a baseline HAI titer of <10 and a post vaccination titer of ≥ 40; or a baseline HAI titer of ≥ 10 and a ≥ 4 fold increase post vaccination (6). Using HAI response as a correlate of protection, we will also be able to estimate the duration of protection and correlate immunologic outcomes with influenza-associated ARI and SARI among the elderly.

Cost data: Records from the Provincial Medical Office and the National Health Services Office will be used to determine the cost of the vaccination campaign, which will be converted into a per capita cost per vaccinee. Data from the Provincial Medical Office will also be collected on average cost of outpatient visits for acute respiratory infection and inpatient admissions for pneumonia. We will also account for indirect costs of missing work days for elderly and their caregivers, and transportation cost. This information will be used to determine the cost-effectiveness of IIV3 among the elderly.

Functional Status:

Participants will be questioned at enrollment about their functional status along with other baseline medical conditions such as co-morbidities using a standardized questionnaire. Functional status is the individual's ability to perform normal activities of daily living. We will use the index from the Vulnerable Elders Survey (7) along with individual elements from other indices and test their association with ARI to determine if experiencing ARIs or SARIs impacts functional status. Cognitive function will be self-assessed as other measures require trained medical personnel to implement. Vulnerable Elders Survey (VES-13) is a function-based questionnaire that was developed to screen elderly 65 years of age or greater in the community to determine their risk for health deterioration (7, 8). The questionnaire asks elderly to self-report their age, health, limitations in physical function and functional disabilities, and takes an average of less than five minutes to complete(7, 8). This tool can be administered over the phone or in person. Each response to each question on the form has a point value attached ranging from 0 to 3 points, and it is scored by adding up points based on the responses received, up to 10.(8) Scores >=3 indicate frailty associated with increased mortality. The test has been shown to be reliable (7, 9, 10). The same functional status measurements were used in a companion study which assessed whether functional status correlated with obtaining flu vaccine and has already been translated into Thai. Using the same functional status measurement tool in this study will allow us to compare outcomes between both studies and build upon prior conclusions. Participants will be asked to re-take the functional status VES-13 questionnaire every six months during the study period to measure changes in functional status over time.

In order to measure the impact of the first episode of ARI or SARI on functional status, we will conduct a nested case-control study of participants recovering from an ARI or SARI. Participants who experience their first ARI or SARI after enrollment will be matched with cohort participants who have not yet experienced an ARI or SARI based on age (+/- 5 years), sex, influenza vaccination and most recent functional status measurement. Interviews will be conducted three to four weeks after the onset of ARI or SARI in the case and both cases and controls will be compared with respect to changes in functional status measurement (VES-13 score>=3).

Elderly with COPD: In the standardized baseline questionnaire assessing functional status and medical conditions, participants will be asked whether they have been diagnosed with COPD. If they do, at the end of two years, the number of outpatient visits and inpatient admissions for exacerbations of COPD will be determined (by directly observing their medical records). A COPD exacerbation is defined as "a sustained worsening of the patient's condition, from the stable state and beyond normal day-to-day variations that is acute in onset and may warrant additional treatment in a patient with underlying COPD" (11). We will use the collected nasal self-swabs or healthcare worker collected nasopharyngeal swab taken when the participant was experiencing an ARI or SARI to confirm whether the COPD exacerbations the participant experienced were influenza-associated. At the end of the study, the incidence of all-cause COPD exacerbations and laboratory confirmed influenza-associated COPD exacerbations among the elderly with COPD who fit the ARI or SARI case definitions will be calculated. The incidence among those who were vaccinated will be compared to those who were not vaccinated.

Pilot studies: Vaccination coverage of persons aged 65 years and older in Nakhon Phanom is low, and targeted towards the elderly who also have underlying chronic conditions. The Provincial Medical Office undertook a pilot study in four districts from June-November 2014 to evaluate strategies to increase the vaccination rate and identify factors, such as functional status and chronic medical conditions that are significantly associated with vaccination. The information gained from this study will allow increased precision in sample size estimates and improved measurement of potential confounders. Another pilot study was conducted from February to March 2015 that studied the acceptability, accuracy and validity of taking nasal self-swabs. Of the total 108 participants in the study, all 108 participants (100%) felt that taking the nasal swab by themselves was acceptable, Sixteen participants (15%) felt uncomfortable taking the swab by themselves; however, 107 participants (99%) felt that nasal self-swabbing was easy to perform. Out of the 109 nasal self-swab samples collected, 106 (97.2%) were able to detect RNaseP<37, an indicator that the samples were taken accurately. We are still in the process of analyzing the validity of the data collected.

3.2 Participants

3.2.1 Population participating in the study

The study will be conducted among males and females aged 65 years and older; this population is recommended for influenza vaccination in Thailand. Data from Thailand and other countries have shown that this age group is at increased risk of severe morbidity and mortality due to influenza-associated ARI.

3.2.2 Expected benefit for participants

There are no expected benefits for participants who enroll in the study.

3.2.3 Possible risk if participate in the study

The risks associated with this study are minimal. There is a very minimal risk of abrasion from improperly taking a nasal swab. Risk will be further minimized by a standardized approach to teaching participants how to safely self-swab. Participants may experience discomfort during collection of nasal and nasopharyngeal swabs, and blood samples, as part of study procedures. Participants may also experience mild redness or soreness at local site of blood draw.

While personally identifiable information will be collected for this study, all data collected will be kept in locked cabinets that only study personnel will have access to. The study team will take all possible steps to minimize risks.

3.2.4 Process for obtaining consent

Written informed consent will be sought from the participants of the community study. Community consent will be sought from all village leaders of the selected sub-district for consent to include their village in the study. Additional consent will be taken in the second year for the immunogenicity sub-study.

3.2.5 Participants' protection

In order to protect the participants, all personally identifiable information obtained from the study will be kept on encrypted and password-protected computers or locked within the study offices in Nakhon Phanom and Nonthaburi and will only be available to staff involved in the study. Personally identifiable information will be stored separately from study information and linked by a unique study identification number. Information that leaves the study offices in Nakhon Phanom will have no personal identifying information linking the participants to either the study or their responses.

4. Introduction and background

4.1 Importance and reasons for conducting study in human subjects

Influenza vaccination is recommended for all persons ≥ 65 years. Ongoing monitoring and evaluation of this program is important in order to document the effects of the vaccine and justify the continuation of the program. Critical outcomes to monitor include vaccine uptake, cost and efficacy. Because vaccine is recommended, it is not ethical to do a randomized controlled trial. Using an observational cohort study, we will be able to generate high quality data on VE in this age group, improving on previous studies by using a population-based cohort and confirming influenza infection through use of molecular diagnostics. Information from a concurrent study of predictors of vaccine coverage will provide information on potential confounders of the association between vaccination and disease reduction, improving the validity of VE results. Additionally, data on cost will be collected to determine value for money of the vaccination campaign in preventing cases and additional health care costs.

4.2 Literature review

Elderly patients are at increased risk for severe complications and mortality due to influenza infection. The elderly, defined as adults ≥ 65 years, are at an increased risk for influenza-associated hospitalizations and death compared to adults <65 years of age(12) . The elderly are more susceptible to experiencing complications of influenza-pneumonia which include the exacerbation of underlying medical conditions such as cardiac health and lung

disease (13). Between 2005 and 2008, influenza-pneumonia was responsible for approximately 36,413 annual hospital admissions and 322 in-hospital deaths in Thailand (13). During this same time, the annual incidence of influenza pneumonia in Thailand was highest in patients 75 years of age or older (13). Complications due to influenza also have the potential to decrease functional status and quality of life in the elderly (14). In order to prevent influenza morbidity and mortality, Thailand's Ministry of Public Health (MOPH) began a vaccine campaign in 2009 to vaccinate high-risk influenza groups including adults ≥65 years of age with IIV3.

Studies among adults <65 years of age and children have shown vaccine efficacy and effectiveness in preventing influenza. A 2012 meta-analysis found a vaccine efficacy ranging from 51% to 67% among adults <65 years for laboratory-confirmed influenza, and a large multi-site observational study found VE for laboratory confirmed influenza ranging between 54% and 66% among children 6 months to 8 years in 2010-2011 (15). Elderly persons are known to mount a less robust immune response to vaccines in general, and lab-confirmed influenza VE among the elderly is generally lower than that seen for adults and children. A large randomized efficacy study reporting vaccine efficacy against lab confirmed influenza for adults ≥60 found a range between 26% and 77% in a season when circulating influenza strains were well-matched to the vaccine(15). Data on the effectiveness of the seasonal, trivalent IIV in Thailand are scarce. Among the elderly, only three studies have been conducted in Thailand: one found that IIV reduced hospitalizations among adults ≥ 50 years due to laboratory-confirmed influenza-associated ARI by 47% using a test-negative case control approach that included only hospitalized patients (12); in the second, a randomized, controlled trial, IIV prevented 56% of influenza-like illnesses among adults ≥60 years, although these were not laboratory-confirmed influenza cases (16). The third study, a nonrandomized, controlled, prospective cohort study conducted in Bangkok found a 47.6% vaccine effectiveness rate and about half reduction in incidence of influenza like illness (ILI) in adults ≥60 years that were vaccinated compared to elderly that were not vaccinated, but major limitations to this study and its results include non-randomization as well as non-laboratory confirmed influenza(17). The studies that show hospital and mortality reduction among the elderly who were vaccinated are mostly retrospective and do not have laboratory confirmed influenza data (15).

Immunogenicity studies have been used to test the antibody response of influenza vaccines by quantifying the amount of antibody seroconversion and duration of humoral immune

response post influenza vaccination, and results have been inconsistent among elderly (15). A literature review of 31 studies between 1986 and 2002 comparing influenza antibody response between adults >58 years of age and adults between the ages of 17 and 59 found that the elderly had overall 2 to 4 times lower rates of seroconversion compared to younger adults(18). Another literature review published in 2008 showed that rapid decline of antibody response among elderly within months of receiving the influenza vaccine was not correlated with age(19). Some studies conducted among children and adults suggest that prior influenza vaccinations and higher levels of preexisting serum HAI antibodies may decrease immunologic response to subsequent vaccination (20-22). Confounding between age and number of prior vaccinations could explain a lower serologic response to vaccination in older adults. A longitudinal cohort study measuring trivalent vaccine effectiveness using laboratory confirmed influenza and measuring humoral immune response among the elderly will lead to more definitive answers.

Elderly with certain underlying medical conditions, such as chronic obstructive pulmonary disease (COPD), are especially at high risk from influenza. COPD is a respiratory illness characterized by irreversible airflow obstruction. In 2010, COPD was the sixth leading cause of death in Thailand (23), and in 2002, the prevalence of COPD among the elderly around Bangkok was 7.11 percent(24). Influenza exacerbates COPD symptoms, and vaccinating individuals with COPD can reduce the risk of hospitalizations, morbidity and mortality (25). One study from the United Kingdom showed that influenza vaccination reduced the risk of all-cause mortality in COPD patients by 41% between December and March(26). Another study conducted in the United states among elderly persons with COPD found that the risk of hospitalization due to pneumonia or influenza was reduced by 48% from influenza vaccination (27). A study conducted in the United States among elderly persons during the influenza season found hospitalization costs due to acute and chronic respiratory conditions to be 42% lower in those that were vaccinated (28). A cost effectiveness analysis in Thailand found that depending on the severity of COPD, for every 100 persons with COPD that receive the influenza vaccine, roughly 125,629 to 680,647 Thai Baht was saved (29, 30). To date, there is only one study that examined vaccine efficacy among persons with COPD who were mostly elderly in Thailand. This randomized controlled trial of the efficacy of IIV3 among persons with COPD in Bangkok, Thailand, to prevent influenza-associated ARI found a vaccine effectiveness (VE) of 76% (30). Because the Thailand MOPH is prioritizing adults with COPD to be vaccinated with influenza, and the

elderly with COPD are at heightened risk, more research is needed to confirm the vaccine effectiveness of trivalent IIV3 influenza vaccine among the elderly with COPD in Thailand to support future protocols.

Data on the cost-effectiveness of vaccinating the elderly is also scarce and still needs to be established in Thailand. Because the elderly are at highest risk for costly hospitalizations due to influenza, vaccinating the elderly has shown to be cost-effective across many Western parts of the world (31). Establishing the cost effectiveness of vaccinating elderly in the subtropics has been more difficult, possibly due to the variable seasonality of influenza in the area (31). Despite evidence that influenza costs Thailand between 23.4 and 62.9 million US dollars annually in economic losses (32), and data showing that IIV3 is cost effective for COPD patients, at least one study has suggested that vaccination in the elderly may not provide sufficient value for money (16). This study found that vaccination significantly reduced the incidence of influenza by about half, but did not reduce the incidence of influenza like illness (ILI) nor the cost associated with treatment of ILI (16). In this study, though, none of the participants acquired pneumonia or needed hospitalization during the study period (16). Further data on the cost-effectiveness of IIV3 for persons aged 65 years or older in Thailand are needed to support increases in coverage.

Functional status encompasses the ability to execute self-care, self- maintenance and perform physical activities. Having ARIs or SARIs may impact the short-term and long-term functional status of the elderly, and vaccinating elderly with influenza to reduce the number of ARIs and SARIs they experience could positively impact their functional status (14). On the other hand, studies have shown that functional status of elderly can impact efficacy of influenza vaccination (33, 34). Lower levels of functional status may be associated with a higher risk of death and a lower probability of influenza vaccination, thus, functional status may confound the association of influenza vaccination and risk of all-cause mortality in the elderly (35). Functional status is being recognized as a potential confounding factor in many influenza vaccine effectiveness studies (35-40). By recording functional status, it can be adjusted for as a confounder when calculating vaccine effectiveness, and it can be used to determine if there is an association between experiencing ARIs and SARIs and having a change in functional status among the elderly.

While influenza A is the main cause of respiratory infections in older adults, other viruses present similarly and also contribute to burden (41). Respiratory syncytial virus (RSV) causes lower respiratory lung infections including pneumonia, most commonly in young children (42). There is little information available on the incidence of RSV among older adults and the elderly, and even less on the elderly in Thailand. In the United States, an estimated 9,812 deaths occur annually due to RSV among the elderly and over 78% of RSV-associated underlying respiratory and circulatory deaths occurred in adults 65 years of age or older(43). A prospective cohort study following high risk adults and elderly patients from Rochester, New York, found that RSV resulted in 10.6 percent of the hospitalizations for pneumonia and 11.4 percent for COPD (44). This study also found that patients who were hospitalized for RSV had similar lengths of stay, use of intensive care unit, and mortality to those hospitalized due to influenza A (44). In a study in Thailand between 2008 and 2011, 3.8% of hospitalized adults ≥ 65 years with acute lower respiratory infection (ALRI) tested positive for RSV (42). The same study found that RSV results in about 130 ALRI hospitalizations for every 100,000 elderly adults infected (42). According to another study in Thailand, 2.9% of adults ≥ 50 years who were hospitalized for pneumonia tested positive for RSV (41). While these studies examine the incidence of RSV-associated hospitalizations, they do not account for the burden of RSV-associated ARI in the community in Thailand, for which no data are currently available. Symptoms of RSV are similar to influenza and include fever, runny nose, cough and decreased appetite (45). RSV, like influenza, can be identified using rRT-PCR. Because participants in the study will already have respiratory swabs taken when experiencing ARI or SARI (symptoms related to both influenza and RSV), we will test for both viruses to add on to our current knowledge of RSV incidence among the elderly and determine the burden of RSV in both the community and hospital settings.

4.3 Benefits of the study

The lack of vaccine effectiveness data among elderly in Thailand may be contributing to the low vaccine coverage in the elderly despite recommendations. Only 14-15% of adults ≥ 65 years had been vaccinated against influenza between 2010 and 2012 (46). A longitudinal cohort study assessing IIV3 effectiveness against laboratory-confirmed influenza and the duration of the humoral immune response after subsequent vaccinations will help inform policy. It will also help

close current gaps in knowledge regarding IIV3 effectiveness among the elderly and its cost effectiveness. This study will also determine the incidence of influenza-associated exacerbations of COPD among elderly participants having ARIs in the community and whether IIV3 helped alleviate them and reduced hospitalization rates. Our study will also determine if ARIs and SARIs are associated with a long term negative change in functional status among the elderly. Finally, through this study, the incidence of confirmed-RSV among elderly experiencing ARI or SARI in Thailand will be calculated so that prevention and treatment of RSV among elderly can be reevaluated.

5. Objectives

1. Primary Objectives

1.1: Measure the incidence of laboratory-confirmed influenza-associated ARI and SARI among a cohort of persons aged 65 years and older in 2 districts in Nakhon Phanom Province and calculate vaccine effectiveness by comparing incidence between persons who did and did not receive IIV3, controlling for potential confounders.

1.2: Assess the medical costs (direct and indirect) associated with influenza infections among the elderly and estimate the savings from vaccination based on varying levels of vaccine coverage, and evaluate the value for money of the influenza vaccine campaign program for persons aged 65 years and older

1.3: Among persons aged 65 years and older with COPD, measure the incidence of influenza-associated COPD exacerbations meeting ARI or SARI case definitions, and all-cause exacerbations and compare incidences between vaccinated and unvaccinated persons

2. Secondary Objectives

2.1: Measure the effect of SARI and ARI on change in functional status

2.2: Evaluate duration of humoral immune response of IIV3 among persons aged 65 years and older; and compare difference in humoral immune response by number of past influenza vaccinations

2.3: Measure the incidence of respiratory syncytial virus in persons ≥ 65 years

6. Location and Duration

6.1 Location:

The study will be conducted in That Phanom and Plapak districts in Nakhon Phanom (NP) Province. The data that are collected will be analyzed at the Public Health Office in Muang District, Nakhon Phanom and at the MOPH in Nonthaburi. Dr. Kriengkrai Prasert from the Nakhon Phanom Public Health Office will be the primary researcher for this project. Members of the Thai Ministry of Public Health (MOPH) will be collecting and analyzing the data and laboratory specimens with help from the staff at Thailand MOPH – U.S. CDC Collaboration (TUC). Centers for Disease Control and Prevention (CDC) will work to aid MOPH in their scientific methods of the study.

6.2 Duration:

We will begin recruiting and training health care workers on data collection and laboratory technique in February of 2015 until April 2015 allowing three to four months. Participants enrolled in the study will be followed for two years from June 2015 through June 2017. Patients will be followed for two years as the incidence of influenza and the effectiveness of the annual vaccine composition can vary from year to year, making conclusions from two years more reliable than one alone. Additionally, approximately six more months will be needed for data analysis. The entire study will take place for approximately 2 years and 10 months, and should be completed by December 2017.

7. Research Methodology

7.1 Population

The target population for this study is the elderly (aged 65 years and older) in the That Phanom and Plapak districts of Nakhon Phanom Province.

7.1.1 Sex

Both male and female participants will be included in the study in the proportion they are found in the general population.

7.1.2 Age

The participants must be at least 65 years of age as of 15 May, 2015 to qualify for the study.

7.1.3 Characteristics (i.e. occupation, social status)

Socio-economic status including occupation, level of education, and wealth will not influence study participation.

7.1.4 Illness or symptoms

Non-institutionalized participants will be enrolled regardless of health status (unless a severe acute infection or medical condition would impair their ability to participate).

7.1.5 Number of participants

<u>Sample size for cohort:</u> The cohort sample size is calculated assuming a 50% vaccination rate, 80% power, a probability of committing a type I error of 5% and an incidence of influenza-associated ARI of 6% per year. With an expected VE of 40% based on our population's age and past studies (12, 16, 17), the required sample size is 2,724. Given a 15% loss to follow-up, a total of 3,133 persons should be enrolled, and assuming a 10% refusal rate, a total of 3,446, or, for simplicity, 3,500 individuals need to be selected

These sample sizes were calculated in OpenEpi.com(47) using the Fleiss Method with a correction factor as follows:

Fleiss Method:

$$n_1 = \frac{\left[Z_\alpha\sqrt{(r+1)\overline{pq}} + Z_{1-\beta}\sqrt{rp_1q_1 + p_2q_2}\right]^2}{r(p_1 - q_1)^2}$$

$$n_2 = rn_1$$

where n_1 is the number of cases and n_2 is the number of controls, $\bar{p}$ is the average exposure in the population, $\bar{q}$ is 1-$\bar{p}$, p_1 is the proportion of cases with the exposure, q_1 is 1- p_1, p_2 is the proportion of controls with exposure and q_2 is 1- p_2.

To account for the use of a normal distribution to approximate a binary function, a continuity correction is applied to n_1 as follows to calculate the final sample size, n_{cc}:

$$n_{1cc} = \frac{n_1}{4}\left[1 + \sqrt{1 + \frac{2(r+1)}{n_1 r\,|p_2 - p_1|}}\right]^2$$

$$n_{2cc} = rn_{1cc}$$

where n_{1cc} and n_{2cc} are the corrected sample sizes of n_1 and n_2, respectively.

Sample size for changes in functional status related to ARI or SARI: We will select matched pairs and compare functional status (measured on a 10 point scale by the VES-13) three-four weeks after the onset of an influenza-associated ARI or SARI to a control who has not had an ARI or SARI. As functional status is measured on continuous scale of 1-10, a matched dependent *t* test is used to compute sample sizes. We have used the PROC POWER procedure in SAS (http://support.sas.com/documentation/cdl/en/statug/67523/HTML/default/viewer.htm#statug_power_overview.htm) to calculate the required sample sizes. Assuming a mean score of 4 for the controls and 5 for the cases (higher VES-13 index indicates lower functional status), and standard deviation of 3 for the difference between the two means, a type I error rate of 0.05, correlation of 0.2 and power of 80%, we require 115 in matched pairs (230 in all).

Sample size for seroconversion: Seroconversion for secondary objective 2.2 will be assessed in a sub-sample of participants. We expect seroconversion rates of 30-50% among those vaccinated. In order to calculate a proportion of 50% plus or minus 5%-pts, we need 384 individuals who intend on becoming vaccinated. We will enroll them sequentially.

$$\frac{[Np(1-p)]}{d^2/1.96^2\ (N-1)+p(1-p)}$$

Where *N* is the population size, *p* is the estimated proportion, *d* is the absolute precision. In this study, *N* is the size of the elderly population in the two districts, approximately 15,000; *p* is 0.5 and *d* is 0.05.

7.2 Inclusion Criteria

Elderly who are enrolled in the primary objective community study are also eligible to join the secondary objective 2.2 immunogenicity study and vice versa. The two studies are not required to be mutually exclusive.

For primary objectives, inclusion criteria include:

- Both males and females aged 65 years old or more;
- Non-institutionalized;
- Regular resident of That Phanom or Plapak Province (defined as being regular resident since Songkran 2014)
- Must be able to communicate adequately (able to see, hear and understand questions, and respond appropriately)

For secondary objective 2.2 (immunogenicity)

- In addition to the primary objectives' inclusion criteria, patient must present at the health facility requesting vaccination against influenza (so we can obtain baseline HAI titer).

For secondary objective 2.1 (functional status)

- Same as the primary objectives' inclusion criteria

For primary objective 1.3 (COPD exacerbations)

- In addition to the primary objectives' inclusion criteria, participant must be diagnosed with COPD

For secondary objective 2.3 (RSV incidence)

- Same as the primary objectives' inclusion criteria

7.3 Exclusion Criteria

For primary objectives exclusion criteria include:

- Elderly with body disabilities that may have impact on self- nasal swab or telephone interview (i.e. elderly with stroke)
- Elderly with history of tumor in the nose or nearby areas
- Elderly with history of bleeding order (i.e. hemophilia)
- Elderly who often gets nosebleeds
- Any elderly with an acute medical condition or illness that could not participate in the study

For secondary objective 2.2 (immunogenicity)

- In addition to the primary objectives' exclusion criteria, any participant who has already been vaccinated with an influenza vaccine in 2015
- Known allergy to influenza vaccine or to egg
- Any participant with any medical condition that could prevent them from having a blood draw

For secondary objective 2.1 (functional status)

- Same as the primary objectives' exclusion criteria

For primary objective 1.3 (COPD exacerbations)

- Same as the primary objectives' exclusion criteria

For secondary objective 2.3 (RSV incidence)

- Same as the primary objectives' exclusion criteria

7.4 Discontinuation Criteria

Participants in the community study, who choose to withdraw from the study, move permanently outside of the selected sub-district during the study or die during the study period will be considered discontinued. The study will be terminated if any major event results in the inability to contact majority of study participants or to collect adequate data and keep the data safe.

7.5 Methodology

7.5.1 For primary objectives and secondary objectives 2.1, 2.3

A list of all persons aged 65 years and older will be obtained for all villages in the selected That Phanom and Plapak districts. This list will be constructed by carefully surveying each participating village by health volunteers to get an accurate up-to-date listing of each elderly resident's name, address and age (as listed on their Identification card). Residents will be ordered and sorted in this order: district, sub-district, village, 5-year age band and systematically sampled proportionately using a sampling interval calculated from the total number of residents and the desired sample size, with a random start. A health volunteer and study team member will be paired together to approach their randomly assigned households every day to complete consent and enrollment. Health volunteers will be trained to seek consent for the study and explain the study methods to the participants. The study team will be trained on enrolling participants once they have consented by issuing a standardized baseline study questionnaire to each participant who consents to enrollment. Every participant that enrolls will receive a unique identification number which will be used in place of any identifying information in the central database. Consent and enrollment questionnaires with the elderly will take place in their homes and will take approximately 30 minutes to administer. Afterwards, the trained study staff member will show a standardized video to all enrolled participants on how to self-swab and store the swab correctly as shown in Appendix 5a.

All participants will be provided a self-swabbing kit from the study team that includes Dacron swabs and test tubes with transport media (TM) which are already labeled with the participants' hospital number, along with written instructions. Participants will be asked to take one nasal self-swab, and to place it in the refrigerator in a separate test tube filled with 1-3ml of TM. Participants will then be asked to describe the procedure to ensure they understand the full technique and will be guided appropriately. Participants will be given a study card with their study ID number and assigned health volunteer's telephone number. Participants who experience an ARI, defined as new onset of cough or worsening of chronic cough, will be asked to take a self-swab as soon as possible and then to call their assigned health volunteer to make an appointment to have their swab collected and answer some questions for the ARI/SARI Episode Questionnaire at the time of the appointment (which will be within 24 hours). The assigned health volunteer will bring an ice box and new Dacron self-swab kit to the sick participant's home, and the specimen will be transported in the ice box by the health volunteer to the sub-district health center for temporary storage. Health volunteers will also conduct the Functional Status Questionnaire three to four weeks after the onset of a participant's ARI or SARI and the Outcomes and Costs of ARI Questionnaire 1 week after the participant recovers which will be monitored through the weekly calls and by the study team. If a participant is very ill due to an ARI and needs assistance in taking a nasal self-swab, they can ask their assigned health volunteer for help in obtaining the specimen. Health volunteers will also be trained to contact their assigned participants weekly to identify ARI events that they have not been notified of already and to take a weekly questionnaire. They will attempt three contacts, by phone or in person, at different times of the day before considering the participant to have missed the week. In the case of an ARI event, participants who have not yet taken a self-swab will be asked to self-swab immediately.

For any participant that does not have a phone or for participants who have a phone without a working signal, we will have the health volunteer follow-up with them on a weekly basis by making household visits instead of a weekly call and rely on this method for collection of all data. If a participant without a working phone develops an ARI or SARI during the week and is able to send someone to the health volunteer to let them know, the health volunteer can also follow-up with the patient using the electronic ARI/SARI Episode Questionnaire, but this is not mandatory for participants without a working phone. If the participant needs to get in touch

with the researcher, the health volunteer will be trained to use their phone to call the researcher for them. When health volunteers transport the specimens to the sub-district health center/district hospital, they will place them inside a refrigerator with a controlled temperature of 2-8^{0}C. Research nurses at the district hospital will be trained to transport the swabs from the refrigerator into the liquid nitrogen tank once a day to keep the specimens below -70^{0}C until transported to NIH.

One research nurse from each health center will be responsible for the enrolled participants within their sub-district. In the event that a participant becomes very ill and goes to the hospital or sub-district health center for treatment of an ARI or SARI, the research nurse at the sub-district health center or district hospital will collect a nasopharyngeal swab from the participant and store it in a refrigerator as outlined in appendix 5a. They will administer the ARI/SARI questionnaire (for health centers) and call the study team to notify them about the participant's visit. The study team will inform the participant's assigned health volunteer so the health volunteer can monitor the participant and take the Functional Status Questionnaire and the Outcomes and Costs of ARI Questionnaire after the participant recovers at the appropriate time period. The nasopharyngeal swabs will also be placed inside a liquid nitrogen tank at the sub-district health centers and district hospitals once a day by trained study team personnel. The nurses at the district hospitals will also collect a nasopharyngeal swab for all participants with COPD who come to the COPD clinic due to an exacerbation.

The nasal and nasopharyngeal swabs will be picked up from all health centers involved in the study on a weekly basis by hired study staff and transported to the NIH laboratory in Nonthaburi in a liquid nitrogen tank for testing. They will be tested by rRT-PCR for the presence of ribonuclease P (RNase P). RNase P is commonly used as an internal control in PCR to indicate the presence of human cells. rRT-PCR involves using the nasal swab samples placed inside VTM and mixing them with a buffer before being incubated at 70 degrees Celsius. Then the RNA is extracted from the mixture using Nucleospin RNA virus. The RNA is eventually mixed with a diluted solution of primers and probes and placed onto a PRC plate or in a PCR tube with a mixture of reagents. This PCR tube or plate is then placed inside a Realtime PCR machine at different temperatures and for various time periods. The results are converted to cycle thresholds (4). The cycle threshold number in rRT-PCR indicates how fast the target is detected and tends to correlate with the amount of the target detected. We will consider samples with

cycle thresholds <37 for RNase P to be adequate (4). We will also record the cycle threshold for additional classifications. The results of the PCR testing (whether the participant has influenza or RSV) will be added to the centralized database in Nonthaburi.

As mentioned above, health volunteers will be trained to make weekly visits/calls to the homes of the participants and answer a short electronic questionnaire case report form (CRF) accompanying the visit/call about each participant's incidence of ARI which will be collected on a hand held computer and synced into a centralized data base at That Phanom and Plapak district hospitals at least once a week.. They will also be asked to assess the elderly participant's functional status and cost of having an ARI after any participant recovers from an ARI/SARI at the specific time period; information will be recorded onto a questionnaire CRF on a hand-held computer as well. CRFs will have each participant's unique identification number input and the health volunteer will transfer data regularly to the district hospitals and the centralized database.

All persons with COPD are required to report to the district hospital's COPD clinic if they are experiencing exacerbations and for check-up. If a participant has COPD, the participant's medical records will be checked by a research nurse at each district hospital to record when and the number of times the participant visited a health center to seek care for a COPD exacerbation onto an electronic questionnaire CRF. We will know if the COPD exacerbation was laboratory-confirmed influenza-associated through results of the nasopharyngeal swab testing during their COPD clinic appointment at the same time period and whether patient was experiencing an ARI or SARI.

For primary objective 1.2 (Direct and Indirect costs)

When a participant becomes ill due to an ARI or SARI, we will train health volunteers to ask them about their costs associated with the infection with an electronic questionnaire CRF 1 week after recovering from their illness. If the participant went to a public health center or hospital, staff authorized by the hospital director will check their medical records to determine the cost of the visit. If the participant went to a private clinic, the standardized questionnaire will ask about the amount of money they and their insurance spent in order to treat/get treated for their illness including all laboratory and examination tests, clinician fees, hospital fees, medication fees. The questionnaire will also ask all participants regarding cost of over-the-

counter medications including herbal or traditional medicine costs, transportation fees, and questions to determine how much income their household lost due to missed work days of the elderly participant and of caregivers who take care of the participant to calculate indirect cost.

Records from the Provincial Medical Office and the National Health Services Office will be used to determine the cost of the vaccination campaign, which will be converted into a per capita cost per vaccinee. Data from the Provincial Medical Office will also be collected on average cost of outpatient visits for acute respiratory infection and inpatient admissions for pneumonia.

For secondary objective 2.1 (Functional status)

Participants will be scored on their VES-13 results every six months. The Vulnerable Elders Survey (VES-13) is a function-based questionnaire that was developed to screen elderly 65 years of age or greater in the community to determine their risk for health deterioration (7, 8). The questionnaire asks elderly to self-report their age, health, limitations in physical function and functional disabilities, and takes an average of less than five minutes to complete(7, 8). This tool can be administered over the phone or in person. Each response to each question on the form has a point value attached ranging from 0 to 3 points, and it is scored by adding up points based on the responses received, up to 10.(8) Scores >=3 indicate frailty associated with increased mortality.

In order to measure the impact of the first episode of ARI or SARI on functional status, we will conduct a nested case-control study of participants recovering from an ARI or SARI. Participants who experience their first ARI or SARI after enrollment will be matched with one cohort participant who has not yet experienced an ARI or SARI based on age (+/- 5 years), sex, influenza vaccination and most recent functional status measurement. Interviews will be conducted three to four weeks after the onset of ARI or SARI in the case and both cases and controls will be compared with respect to changes in functional status measurement using a multinomial logistic regression to control for other potential confounders. Beginning from January 2016, when a complete dataset for vaccination and functional status will be available, these interviews will be conducted until the required sample size of 115 matched pairs of cases and controls are met.

For secondary objective **2.2 (Immunogenicity)**

Elderly who have not yet been vaccinated, but have presented to the district hospital for vaccination will be asked by trained study staff at the district hospital to join the immunogenicity subset of the study regardless of whether they joined the primary study. Elderly will be informed of the objective aim, protocol, risks and benefits of joining the immunogenicity study. If elderly consent, they will be asked to have blood drawn before being vaccinated with the inactivated, trivalent influenza IIV3 vaccine the same day (day 0), and return to the district hospital again to have blood drawn 28 days (1 month), 180 days (6 months) and 360 days (1 year) after receiving the vaccine. One additional blood draw will be requested after 28 days of second vaccination in the year 2 to compare the effect of prior vaccination on level of immunological response. For participants unable to travel to the health center, the research nurse will travel to the elderly participant's home and obtain the necessary blood sample, and transport the sample back to the district hospital in ice packs. Participants will be asked for 5cc of blood per scheduled appointment. The serum will be separated from the blood samples using a centrifuge at the district hospital and stored in a refrigerator between 4-8^{0}C for a week or less. On a weekly basis, the serum samples will be transported in ice-packs for less than 24 hours to the NIH laboratory in Nonthaburi. When participants consent to being enrolled, the study staff that consents and enrolls them will also take an electronic baseline assessment questionnaire CRF that should take about 15 minutes to administer. They will provide the participant with a study ID card which has their individual study number and the days of their next scheduled appointments. The card will also have a number to call a study team member if the participant should need to reschedule their appointment or has any other issues or concerns.

Once the serum samples reach the NIH laboratory, hemagglutination inhibition assay (HAI) will be used to measure influenza antibody count specific to the three strains in IIV3: influenza A H1N1, influenza A H3 and influenza B(5). In order to perform this measurement, serum is first diluted and treated to remove nonspecific agglutinins. Red blood cells (RBCs) are mixed with phosphate buffered saline (PBS) solution and standardized to a 0.5% concentration. Chosen influenza strains are used to create standardized control antigens through titration and back titration(5). Specific amounts of serum and antigen controls are eventually mixed in PBS in a microtitre plate and incubated. RBCs are then mixed in and the mixture is set in room temperature until the RBCs in the mixture settle(5). Once the mixture is settled, the antibodies

are counted. Seroconversion will be measured as the percent of participants who have one of the following: a baseline HAI titer of <10 and a post vaccination titer of ≥ 40; or a baseline HAI titer of ≥ 10 and a ≥ 4 fold increase post vaccination(6).The results of the serum samples will also be recorded into the centralized database by study staff in Nonthaburi.

7.5.2 Participant selection method

Primary study

That Phanom and Plapak Districts have approximately 6,000 and 3,000 elderly members, respectively. There are 136 villages in That Phanom and 85 villages in Plapak. In order to maintain population proportions, approximately 2/3 of the 3,500 calculated necessary sample size will be chosen from That Phanom (about 2,333 individuals) and approximately 1/3 will be selected from Plapak (about 1,167 individuals). To get proportional representation, we will use systematic random sampling. The elderly will be sorted in this order: district, sub-district, village, 5-year age band. The sampling interval will be calculated as the total number of elderly residents divided by the total sample size. A random number will be selected from within the sampling interval as the starting point. The random start plus the sampling interval will be applied to the list to select the sample.

Functional status sub-study

One hundred fifteen elderly with a first ARI or SARI will be interviewed within three to four weeks of the onset of their illness to determine their VES-13 score. A participant who has not had ARI or SARI since the start of the study will be randomly selected from among those who match the case on age (+/- 5 years), sex, influenza vaccination and most recent functional status measurement and interviewed for functional status at the same time as the case.

Immunogenicity study

All elderly that meet the inclusion and exclusion criteria who come to the district hospital to obtain the influenza vaccine beginning from 15 May, 2015 will be asked to join the immunogenicity study. The first 384 elderly who consent will be enrolled in the study. Consent will be sought again from this group at the start of the second year for additional study to compare the difference in immunologic response after subsequent vaccination.

7.5.3 Data collection and tool

For the community study, health volunteers and study team members will be responsible for collecting baseline data from consented participants during enrollment. Baseline data includes questions regarding health and disease states, education and wealth (Appendix 4B) and also people living with the participant (Appendix 4i, which will be collected after approval of the protocol amendment, once at the subsequent health volunteer weekly follow up). Onwards, trained health volunteers are responsible for collecting all data in the community regarding details of new episodes of ARI/SARI on a weekly basis, functional status of participants 3 to 4 weeks after the onset of the ARI/SARI, and total costs of having an ARI/SARI 1 week after the participant recovers (Appendix 4c, 4d, 4f and 4g, respectively). Trained nurses will be responsible for collecting data on episodes of ARI/SARI for participants who come to their health center for treatment after their appointment (Appendix 4e). Participants will be responsible for collecting nasal self-swabs in the community and trained research nurses will be responsible for collecting nasopharyngeal swabs at health centers. The health volunteers and study team members will be responsible for transporting all nasal specimens to NIH in Nonthaburi for analysis. For the immunogenicity study, a research nurse will be responsible for collecting data during enrollment regarding immunity of the participant and collecting all blood draws (Appendix 4h). The study team will be responsible for transporting the blood samples to NIH in Nonthaburi for analysis. All data collected by health volunteers, the study team and nurses (including enrollment questionnaires, weekly questionnaires, and follow-up questionnaires) will be on electronic CRFs on a hand-held computer device and will be transmitted to a central database system on at least a weekly basis by health volunteers and the study team, and on a daily basis by research nurses. Laboratory results of respiratory swabs and blood samples will be entered onto a separate database at the NIH laboratory in Nonthaburi and linked to the central database later on via the unique assigned identification number.

7.6 The process of obtaining informed consent

For the primary study, written informed consent will be sought from the participant at the participant's home by trained health volunteers, prior to enrollment with the presence of a witness. Study staff will also request written informed consent from village leaders in the

selected sub-district for permission for their village to participate. For the immunogenicity study, written informed consent will be obtained from elderly coming for their influenza vaccination at the district hospitals in That Phanom and Plapak by trained study staff and in the presence of a witness.

For the immunogenicity study, a trained research nurse will meet the vaccination team at both district hospitals during regular business hours and during mobile vaccination clinic runs in the community. When an elderly person approaches the vaccination counter at each hospital or mobile clinic, the research nurse will screen him/her to see if he/she is eligible for the study. If eligible, the nurse will read through the contents of the information sheet disclosing all risks and benefits, answer questions the elderly person may have and ask the elderly person if they would like to enroll into the study. The elderly will also be assured that they may refuse to join the study for any reason without any repercussions and will be able to obtain a vaccination either way. If the elderly person agrees, the nurse will be responsible for consenting and enrolling the elderly into the study. Before take blood draw at the follow up 360 days, nurse will do a new consent elderly person for additional study. If the elderly person refuses for any reason or is ineligible, the nurse will direct the elderly person to the vaccination team who will administer the influenza vaccine to the elderly. In instances in which consenting persons are unable to read, health volunteers and/or study staff will read an oral consent script and then collect written documentation of oral consent for study participation. Consent forms are written in English at the Flesch-Kincaid reading levels of 6.9. All consents will be translated into Thai by an experienced translator and checked by a senior Thai scientist who is fluent in both Thai and English languages. These will then be back-translated to ensure accuracy.

Personnel enrolling individuals into the study will be trained to ensure that potential participants feel no pressure to join the study and understand that enrollment is optional.

The study participants will be clearly notified of the purpose of the study, and what will be required of the participant during the study.

Participants in the study will be notified that there are minimal risks associated with being enrolled in the study.

Participants in the study will be notified that there is no health benefits associated with being enrolled in the study.

Study participants will be allowed to withdraw from the study at any time without or loss of benefits to which they are entitled and notified of such.

7.7 Data Collection

The baseline data from enrollment will be collected on electronic CRFs by the study team and transcribed to a centralized database on a daily basis. Weekly assessments and all follow-up questionnaires will also be collected by health volunteers on electronic CRFs and the responses will be transmitted to the central database at least once a week at the district hospital. When a participant is admitted to the hospital, a research nurse will look at patient charts to inquire reasons for admit and take a nasopharyngeal swab if applicable. All relevant data collected during hospitalization or clinic visits will also be recorded on electronic CRFs, and transmitted to the central database on a daily basis at the district hospital. Laboratory results of respiratory swabs and blood samples will be entered onto a separate database at the NIH laboratory in Nonthaburi and linked to the central database later on via the unique assigned identification number.

Any edits made to the original record in the hand-held computer will be noted in a separate database with record of the time and date, identification number of the person making changes and the change that was made. Personal identifying data will be kept separate from the study database, linked by a unique study identification number. At the conclusion of the study, after data have been cleaned, the table with personal identifying data will be deleted. For the purposes of study oversight, CDC investigators will have access to personal identifying information during the course of the study, until the study is anonymized. Laboratory results will be entered into a separate database and linked to the central study database via the unique study identification number, and personal identifying data linked to the laboratory results will be deleted.

7.8 Quality Control

Electronic questionnaires will have built-in ranges and automated checks to decrease opportunities to enter incorrect information. Study personnel will be carefully trained to properly

fill out each questionnaire to improve uniformity and accuracy. The data will be transmitted to the centralized database in a timely fashion so that the data can be monitored throughout the study period for errors and tested prior to use.

Protection of Privacy and Confidentiality

All data will be collected on password protected handheld computers. Personally-identifiable data will be kept separate from study questionnaires and linked only by a study identification number. Project paperwork will remain in a double locked, secure location at the Provincial Health Office once they have been transcribed to the central database, available only to a minimum number of local project staff, and will not be reused or disclosed to any other person or entity except as required by law, for authorized oversight of the research project, or for other research for which the use or disclosure of protected health information would be permitted.

Each community participant will be given a study identification number that can be linked to name only by study staff. All identifiers will be destroyed at the earliest opportunity. Two years after the study results are made public, all records will be destroyed. Data on computers will be permanently deleted. All paper documentation including CRFs and consent forms will be shredded. Nasal and nasopharyngeal swab samples and blood samples will be destroyed after 2 years by being stripped of any identifiers, sterilized by autoclave and thrown away or incinerated.

7.9 Data Analysis

In order to evaluate the primary research question – the vaccine effectiveness of the inactivated trivalent IIV3 vaccine in the elderly - we will use Poisson regression with person-time at risk as an offset. The outcome variable will be the number of influenza associated ARI or SARI events in each given year of life among 2 groups, elderly who have been vaccinated with IIV3 and elderly who have not. The resulting count data will account for multiple outcomes in the same participant. We will also employ GEE methodology to the Poison regression to account for geographical clustering by cases and correlation between outcomes in individuals with repeated measures. We can control for other predictive variables that may be associated with

underlying disease such as age, co-morbidities and distance to health facility. To reduce any potential non-random loss-to-follow up bias, we will use propensity score matching in addition to controlling for confounders. To address the other primary study question regarding cost, we will obtain estimates of direct and indirect costs from each of the groups and apply differences to the adjusted risks to arrive at the adjusted cost differences between elderly who have been vaccinated and elderly who have not been vaccinated.

In order to measure the effect ARI and SARI have on functional status (secondary objective 2.1) we will compare change in functional status among those that did and did not experience ARIs or SARIs by matching participants to one control when they become sick to see if there is a significant difference in functional status measurements. We will use a nested case control study to compare the two groups. By matching those who experienced a first ARI or SARI with those who had not on age (+/- 5 years), sex, influenza vaccination and most recent functional status measurement (+/-1 point), we can account for unknown confounders that may cause a change in functional status while controlling for known confounders.

To estimate the incidence of influenza-associated COPD exacerbations among elderly with COPD, we will calculate the total number of laboratory-confirmed influenza-associated COPD exacerbations per total number of COPD patients within our cohort over a year (primary objective 1.3). We will compare the incidence among those that were vaccinated with IIV3 versus those that were not vaccinated with IIV3. In order to account for correlation among repetitive measurements from each individual, we will also employ GEE methodology. This method will also allow us to adjust for underlying confounders such as smoking history and age.

Blood samples will be taken from a select number of participants who will have had IIV3 to test the antibody response of IIV3 among the elderly (objective 2.2). These blood samples will be taken at baseline before receiving the shot, and then again after 28 days (1 month), 180 days (6 months), and 360 days (12 months) later. The blood samples will be tested for HI antibodies. Everyone with a baseline HI assay ≥10 who has a four-fold increase in HI antibody count post vaccination or who has a baseline HI assay < 10 who has a post vaccination count ≥40 will be considered seroconverted against influenza. One additional blood draw will be taken after 28 days of subsequent vaccination in the second year to compare the effect of prior vaccination on level of immunological response. We will use a random effects model (RE) to compare blood

samples at the post vaccination dates to the baseline HI assay to determine how long participants vaccinated with IIV3 retain an adequate number of HI antibodies. RE allows us to control for the differences in antibody count amongst all individuals across unequal intervals and obtain a mean distribution. This methodology will also allow us to control for confounders such as age and factors which make participants immuno-compromised.

We will also evaluate a simpler outcome, the incidence rate of RSV among elderly with ARI or SARI over the entire observation period (second-objective 2.3), which should be similar between those that were vaccinated with Influenza IIV3 and those that were not.

Aggregate results may be shared with relevant CDC programs in which the findings of this study may have immediate policy implications. Opportunities for publication will be sought.

7.10 Specific consideration

Only 384 of the 3,500 or more total participants enrolled will be participating in second objective 2.2 which involves having blood drawn periodically in order to test serological response to the inactivated trivalent influenza IIV3 vaccine. Each participant will need to have their blood drawn five times: right before receiving the influenza vaccine on day 0, and then on days 28 and 180 and 360 after receiving the vaccine; and one additional time on days 28 of subsequent vaccination in the second year. Each blood draw will be 5cc.

8. Ethical considerations

8.1 Possible risks including preventive or alleviative measures

Based on a review of clinical documents and past study procedures, there is minimal risk from taking a nasopharyngeal swab, especially when medical conditions affecting the nose are not present (48). Participants may experience mild discomfort and experience a gag reflex, an uncomfortable contraction in the back of the throat when areas of the back of the throat are touched. Nasal swabs have similarly been associated with minimal risk of mostly discomfort. According to a study in 2010 asking patients to rate their discomfort in collecting self-swabs,

65% stated mild to no discomfort, 31% reported moderate discomfort, and 4% reported severe discomfort (49). For community members taking their own nasal swab, there is a minimal risk of injuring oneself from swabbing too deep or too forcefully, but this minimal risk is mitigated by the training provided to the community participants prior to self-swabbing.

For the participants who will also be involved in the immunogenicity study component, participants may experience discomfort, mild redness, or soreness at the local site of the blood draw. To mitigate the risk of blood draw, only experienced and knowledgeable professionals will withdraw blood from participants, and they will be able to aid participants should any unanticipated problem arise.

It is possible that the influenza vaccine for the following year will be available before 1 year in the Immunogenicity study is over for our participants. In order to avoid elderly participants from being able to obtain the vaccine the following year, we will notify all participants that they can get the vaccine for the next year when it becomes available, but that they should notify us before getting the vaccine so that we can schedule their last blood draw appointment before they get the vaccine.

8.2 Compensation

The money used to compensate participants is minimal and only available in order to defer the cost of time, transportation, and phone calls so that participating in the study will be neither a burden nor an incentive for making money. All participants will be compensated with a total of 1000 baht per year for their time and phone calls to health volunteers for a total of 2,000 baht for the entire study period. They will initially receive 300 baht upon enrolling in the study. They will receive another 300 baht after 6 months if they are still enrolled, and 400 baht after 12 months. If they stay enrolled for the second year, they will be reimbursed the same way as the first year. If participants need to go to the local health center for a clinical visit, they will be compensated 100 baht for transportation. Participants enrolled in the immunogenicity study will receive 300 baht with each blood draw, so they will get a total of 1,200 baht for the year 1 and additional 300 baht (total 1,200 baht) for year 2 of the immunogenicity study if they remain enrolled. As mentioned and outlined in section 7.8, all participants' personal information will remain confidential and secure.

Estimated Budget and Budget Justification

Total budget: $456,016.13$987097.81 or 32,485,28 314,136,500 Baht (For the first year) 30,644,031.15 Baht for the second year

Justifications: The study is being funded by the Centers for Disease Control and Prevention.

8.3 Other ethical considerations

Because we are a research team conducting an observational study, we will not interfere with participants' decisions to become vaccinated or not because this can bias the results. If a participant is interested in obtaining the vaccine and inquires our research team about the process, however, we will provide objective information about when and where participants can obtain the influenza vaccine if they desire.

8.4 Information sheet

Information sheets have been prepared for village leaders and community participants (Please see Appendix)

Dissemination, Notification, and Reporting of Results

Notifying Participants of their Individual Results

The results of testing will not be done in a timely enough fashion and will not be used to guide treatment decisions, but participants will be provided access to their results. The results of their blood titers, nasal swabs and nasopharyngeal swabs will be linked to the participant's Hospital Number (HN), and the results will be returned to the hospital to be placed in participant's medical records so participants may inquire about them at later visits.

Notifying Participants of Project Findings

Participants will not be notified individually of specific findings of this surveillance project. The results of the study will be disseminated through peer-reviewed scientific journals and become part of publicly available information.

Study Records

Personal identifying information from the community study will be deleted as soon as data cleaning is complete. Study records will be maintained for two years following the completion of the study manuscript. At the end of the two years, records will be destroyed unless they subsequently are used for another IRB reviewed protocol or there is a public health, research or legal requirement to maintain the records. Data on computers and handheld devices will be permanently deleted. Any paper documentation including consent forms will be shredded. Nasal and nasopharyngeal swab samples and blood samples will be destroyed after 2 years by being stripped of any identifiers, being sterilized by autoclave and thrown away or incinerated.

Limitations

The main limitation of this study is its limited generalizability to Thailand. While we are able to obtain a representative sample of Nakhon Phanom Province, the people in NP may be different than people from other parts of Thailand. Another limitation is sampling bias. The study's sample may be biased since healthier people are usually more willing to participate in studies compared to people who are more ill, which would not represent the target population of all types of elderly people. In order to diminish the potential of sampling bias, our inclusion criterion includes all elderly despite illness type.

It is possible that not all respiratory events in the enrolled elderly will be captured, due to inability to communicate, misunderstanding and even memory loss. To a large extent, a lot of the success for this project may rely on the relationship between the household and health volunteer and role of the caregiver for participants greater than 80 years of age. Another limitation to our study is that baseline enrollment data and weekly follow-up data is relying heavily on self-report. It is possible for participants to be unaware of whether they have certain underlying co-

morbidities if they do not regularly visit the physician, it is possible for them to under or overestimate their functional status, and it is possible for them to forget to take a nasal self-swab or remember that they even experienced an ARI or SARI. In order to mitigate these potential issues, we are using a reliable and sensitive functional status questionnaire and following-up with participants weekly in case they forget to call us.

Although Thailand provides free vaccinations for elderly, there are not enough purchased to vaccinate all elderly in Thailand. Due to the limited number of vaccines, elderly that are sicker and see health care professionals more often are more likely to become vaccinated than those who are healthier. The elderly who join our immunogenicity study will most likely be sicker than the general population and are more likely to be immunocompromised. Because of this selection bias, our immunogenicity study will most likely show a lower rate of seroprotection among the elderly than what is actually representative of the average elderly population.

Roles and Responsibilities

The principal investigator, Dr. Kreingkrai Prasert, will be responsible for overseeing all aspects of the study design, ethical review, data analysis, interpretation and manuscript preparation. CDC and TUC will be responsible for ensuring appropriate scientific and ethical review of the protocol. Health volunteers and nurses will be responsible for communicating and enrolling participants, as well as follow-up. MOPH will provide support to the health volunteers and nurses in their roles, prepare and analyze laboratory samples, and merge datasets. Data analysis will be conducted by TUC with guidance from Thammasat University. All co-authors will review and approve the final draft of all publications.

References:

1. Akmatov MK, Gatzemeier A, Schughart K, Pessler F. Equivalence of self- and staff-collected nasal swabs for the detection of viral respiratory pathogens. PLoS One 2012;7(11):e48508.
2. Kim C, Ahmed JA, Eidex RB, Nyoka R, Waiboci LW, Erdman D, et al. Comparison of nasopharyngeal and oropharyngeal swabs for the diagnosis of eight respiratory viruses by real-time reverse transcription-PCR assays. PLoS One 2011;6(6):e21610.
3. Spencer S, Gaglani M, Naleway A, Reynolds S, Ball S, Bozeman S, et al. Consistency of influenza A virus detection test results across respiratory specimen collection methods using real-time reverse transcription-PCR. J Clin Microbiol 2013;51(11):3880-2.
4. CDC protocol of realtime RTPCR for Influenza A(H1N1). In: Prevention CfDCa, editor. 2 ed. Atlanta, U.S.: World Health Organization; 2009. p. 8.
5. Organization WH. Manual for the laboratory diagnosis and virological surveillance of influenza. In. Geneva, Switzerland: World Health Organization Press; 2011. p. i-140.
6. Garg S, Olsen SJ, Fernandez S, Muangchana C, Rungrojcharoenkit K, Prapasiri P, et al. Seroincidence of Influenza Among HIV-infected and HIV-uninfected Men During the 2009 H1N1 Influenza Pandemic, Bangkok, Thailand. Open Forum Infectious Diseases 2014;1(3).
7. Saliba D, Elliott M, Rubenstein LZ, Solomon DH, Young RT, Kamberg CJ, et al. The Vulnerable Elders Survey: a tool for identifying vulnerable older people in the community. J Am Geriatr Soc 2001;49(12):1691-9.
8. Vulnerable Elders Survey. In. Santa Monica, California: Rand Corportation; 2014.
9. Min LC, Elliott MN, Wenger NS, Saliba D. Higher vulnerable elders survey scores predict death and functional decline in vulnerable older people. J Am Geriatr Soc 2006;54(3):507-11.
10. Mohile SG, Bylow K, Dale W, Dignam J, Martin K, Petrylak DP, et al. A pilot study of the vulnerable elders survey-13 compared with the comprehensive geriatric assessment for identifying disability in older patients with prostate cancer who receive androgen ablation. Cancer 2007;109(4):802-10.
11. Burge S, Wedzicha JA. COPD exacerbations: definitions and classifications. Eur Respir J Suppl 2003;41:46s-53s.
12. Dawood F, Prapasiri P, Areerat P, Ruayajin A, Chittaganpitch M, Muangchana C, et al. Effectiveness of the 2010 and 2011 Southern Hemisphere trivalent inactivated influenza vaccines against hospitalization with influenza-associated acute respiratory infection among Thai adults aged â‰¥ 50 years. Influenza and other respiratory viruses 2014;8(4):463-8.
13. Simmerman JM, Chittaganpitch M, Levy J, Chantra S, Maloney S, Uyeki T, et al. Incidence, seasonality and mortality associated with influenza pneumonia in Thailand: 2005-2008. PLoS One 2009;4(11):e7776.
14. Gozalo PL, Pop-Vicas A, Feng Z, Gravenstein S, Mor V. Effect of influenza on functional decline. J Am Geriatr Soc 2012;60(7):1260-7.
15. Centers for Disease C, Prevention. Prevention and control of seasonal influenza with vaccines. Recommendations of the Advisory Committee on Immunization Practices--United States, 2013-2014. MMWR Recomm Rep 2013;62(RR-07):1-43.

16. Praditsuwan R, Assantachai P, Wasi C, Puthavatana P, Kositanont U. The efficacy and effectiveness of influenza vaccination among Thai elderly persons living in the community. J Med Assoc Thai 2005;88(2):256-64.
17. Plasai V, Lertmaharit S, Viputsiri OA, Pongpanich S, Panichpathompong U, Tarnmaneewongse V, et al. Influenza vaccination among the elderly in Bangkok. Southeast Asian J Trop Med Public Health 2006;37 Suppl 3:140-4.
18. Goodwin K, Viboud C, Simonsen L. Antibody response to influenza vaccination in the elderly: a quantitative review. Vaccine 2006;24(8):1159-69.
19. Skowronski DM, Tweed SA, De Serres G. Rapid decline of influenza vaccine-induced antibody in the elderly: is it real, or is it relevant? J Infect Dis 2008;197(4):490-502.
20. Sasaki S, He XS, Holmes TH, Dekker CL, Kemble GW, Arvin AM, et al. Influence of prior influenza vaccination on antibody and B-cell responses. PloS one. 2008;3((8):e2975.
21. Gross PA, Sperber SJ, Donabedian A, Dran S, Morchel G, Cataruozolo P, et al. Paradoxical response to a novel influenza virus vaccine strain: the effect of prior immunization. Vaccine 1999;17(18):2284-9.
22. Beyer WP, Palache AM, Sprenger MJ, Hendriksen E, Tukker JJ, Darioli R, et al. Effects of repeated annual influenza vaccination on vaccine sero-response in young and elderly adults. . Vaccine 1996;14(14):1331-9.
23. Centers for Disease Control and Prevention. CDC in Thailand Factsheet. In; 2014.
24. Maranetra KN, Chuaychoo B, Dejsomritrutai W, Chierakul N, Nana A, Lertakyamanee J, et al. The prevalence and incidence of COPD among urban older persons of Bangkok Metropolis. J Med Assoc Thai 2002;85(11):1147-55.
25. Mowls DS, Cheruvu VK, Zullo MD. Influenza vaccination in adults with chronic obstructive pulmonary disease: the impact of a diagnostic breathing test on vaccination rates. PLoS One 2013;8(6):e67600.
26. Schembri S, Morant S, Winter JH, MacDonald TM. Influenza but not pneumococcal vaccination protects against all-cause mortality in patients with COPD. Thorax 2009;64(7):567-72.
27. Nichol KL, Baken L, Nelson A. Relation between influenza vaccination and outpatient visits, hospitalization, and mortality in elderly persons with chronic lung disease. Annals of internal medicine 1999;130(5):397-403.
28. Nichol KL, Margolis KL, Wuorenma J, Von Sternberg T. The efficacy and cost effectiveness of vaccination against influenza among elderly persons living in the community. N Engl J Med 1994;331(12):778-84.
29. Wongsurakiat P, Lertakyamanee J, Maranetra KN, Jongriratanakul S, Sangkaew S. Economic evaluation of influenza vaccination in Thai chronic obstructive pulmonary disease patients. J Med Assoc Thai 2003;86(6):497-508.
30. Wongsurakiat P, Maranetra KN, Wasi C, Kositanont U, Dejsomritrutai W, Charoenratanakul S. Acute respiratory illness in patients with COPD and the effectiveness of influenza vaccination: a randomized controlled study. Chest 2004;125(6):2011-20.
31. Nichol KL. The efficacy, effectiveness and cost-effectiveness of inactivated influenza virus vaccines. Vaccine 2003;21(16):1769-75.
32. Simmerman JM, Lertiendumrong J, Dowell SF, Uyeki T, Olsen SJ, Chittaganpitch M, et al. The cost of influenza in Thailand. Vaccine 2006;24(20):4417-26.

33. Chan TC, Hung IF, Luk JK, Shea YF, Chan FH, Woo PC, et al. Functional status of older nursing home residents can affect the efficacy of influenza vaccination. J Gerontol A Biol Sci Med Sci 2013;68(3):324-30.
34. Hara Y, Hagihara A, Ikematu H, Nobutomo K. Efficacy of influenza vaccine among elderly patients by physical activity status. Environ Health Prev Med 2002;7(5):183-8.
35. Jackson LA, Nelson JC, Benson P, Neuzil KM, Reid RJ, Psaty BM, et al. Functional status is a confounder of the association of influenza vaccine and risk of all cause mortality in seniors. Int J Epidemiol 2006;35(2):345-52.
36. Babu GB, T. Influenza Vaccination To Elderly: Quantifying The Potential Role Of Unmeasured Confounders Through An Example. The Internet Journal of Epidemiology 2009;9(1).
37. Groenwold RH, Hoes AW, Nichol KL, Hak E. Quantifying the potential role of unmeasured confounders: the example of influenza vaccination. Int J Epidemiol 2008;37(6):1422-9.
38. Landi F, Onder G, Cesari M, Gravina EM, Lattanzio F, Russo A, et al. Effects of influenza vaccination on mortality among frail, community-living elderly patients: an observational study. Aging Clin Exp Res 2003;15(3):254-8.
39. Nichol KL, Nordin JD, Nelson DB, Mullooly JP, Hak E. Effectiveness of influenza vaccine in the community-dwelling elderly. N Engl J Med 2007;357(14):1373-81.
40. Kissling EM, Alain; Valenciano, Marta. Protocol for cohort database studies to measure pandemic and seasonal influenzavaccine effectiveness in the European Union and European Economic Area Member States. In: Control ECfDPa, editor. Stockholm: European Centre for Disease Prevention and Control; 2009.
41. Olsen SJ, Thamthitiwat S, Chantra S, Chittaganpitch M, Fry AM, Simmerman JM, et al. Incidence of respiratory pathogens in persons hospitalized with pneumonia in two provinces in Thailand. Epidemiol Infect 2010;138(12):1811-22.
42. Naorat S, Chittaganpitch M, Thamthitiwat S, Henchaichon S, Sawatwong P, Srisaengchai P, et al. Hospitalizations for acute lower respiratory tract infection due to respiratory syncytial virus in Thailand, 2008-2011. J Infect Dis 2013;208 Suppl 3:S238-45.
43. Thompson WW, Shay DK, Weintraub E, Brammer L, Cox N, Anderson LJ, et al. Mortality associated with influenza and respiratory syncytial virus in the United States. JAMA 2003;289(2):179-86.
44. Falsey AR, Hennessey PA, Formica MA, Cox C, Walsh EE. Respiratory syncytial virus infection in elderly and high-risk adults. N Engl J Med 2005;352(17):1749-59.
45. Oregon Health & Science University. Influenza and RSV. In; n.d.
46. Owusu JT, Prapasiri P, Ditsungnoen D, Leetongin G, Yoocharoen P, Rattanayot J, et al. Seasonal influenza vaccine coverage among high-risk populations in Thailand, 2010–2012. Vaccine (0).
47. Sullivan KMS, Minn M. Sample Size for a Cross-Sectional, Cohort, or Clinical Trial Studies. In; 2007.
48. Nasopharyngeal culture. In: MedlinePlus. Bethesda, MD: U.S. National Library of Medicine, U.S. Health and Human Services; 2014.
49. Smieja M, Castriciano S, Carruthers S, So G, Chong S, Luinstra K, et al. Development and evaluation of a flocked nasal midturbinate swab for self-collection in respiratory virus infection diagnostic testing. J Clin Microbiol 2010;48(9):3340-2.

Appendix

1. Budget
2. Participants
    a. Participant information sheet for VE study
    b. Participant consent form for VE Study
    c. Participant information sheet for Immunogenicity (secondary objective 2.2)
    d. Participant consent form for Immunogenicity (secondary objective 2.2)
    e. Blood draw appointment sheet (secondary objective 2.2)
    f. Participant information sheet for additional immunogenicity study year 2 (secondary objective 2.2)
    g. Participant consent form for additional immunogenicity study year 2 (secondary objective 2.2)
3. Village Leaders
    a. Village Leader information sheet for VE Study including Immunogenicity
    b. Consent form for village leader
4. Questionnaires
    a. Personal Identifying Information, Eligibility and Consent (For Health Volunteer)
    b. Enrollment Questionnaire (For Health Volunteer)
    c. Weekly Contact Questionnaire (For Health Volunteer)
    d. ARI/SARI episode Questionnaire (For Health Volunteers)
    e. ARI/SARI episode Questionnaire (For Trained Nurses in Health Centers)
    f. Functional Status Questionnaire
    g. Outcomes and Costs of Acute Respiratory Illness Questionnaire
    h. Screening and Enrollment Questionnaire for Immunogenicity study
        i. Questionnaire 1: Personal Data
        ii. Questionnaire 2: Screening Form
    i. Number and characteristic of people living with participant
5. Written instructions
    a. Standard Operating Procedure for Nasal self-swabs and Nasopharyngeal swabs

Budget

This study is supported by Thailand MOPH and US-CDC collaboration. The health technical office under the office of permanent secretary of Thai MOPH is the grantee. The budget for the activity in the first year is 987.097.81 USD (32,485,283 baht) as the detail show in the table below;

Item	budget (baht)
1. Personnel	1,568,000
2. Social security	21,000
3. Travel	644,000
4. Office supply	610,000
5. Laboratory supply	3,430,689
6. Cost for hiring research activity conducting	6,322,822
7. Laboratory testing	19,887,972
**Total**	**32,485,283**

The budget for the activity in the second year is estimated at 30,644,013.15 baht as the detail show in the table below;

Item	budget (baht)
1. Personnel	13,573,320.00
2. Social security	218,100.00
3. Travel	1,060,000.00
4. Office supply	254,871.00
5. Laboratory supply	1,200,000.00
6. Cost for hiring research activity conducting	0.00
7. Laboratory testing	14,337,722.15
Total	**30,644,013.15**

2a. Participant information sheet for Vaccine Effectiveness Study

Flesch-Kincaid Grade Level: 6.9

Research name: Flu 8: Measuring the effectiveness of the trivalent inactivated influenza vaccine in persons aged 65 years and older in Thailand

Principal Investigator

Dr. Kriengkrai Prasert Position: Medical physician senior professional level

Place of work: Nakhon Phanom Public Health Office

Tel. Office phone: 042-511 410 Mobile phone: 081-975-5460 Fax 042-512 463

Co-Principal Investigator (Provincial Health Office)

Dr. Preeda Worahan

Tel. Mobile phone: 088-563-7079 and 081-871-9863

Hello. My name is ______________________________. I work for the Ministry of Public Health and the Provincial Health Office. We are doing a large study in Nakhon Phanom. The purpose of this study is to learn how well the flu vaccine works in Thailand to stop people from becoming sick and to save money on treatment. We also want to know how having an acute respiratory infection effects health. We also want to know how often people get sick from the respiratory syncytial virus. This information will help us learn how to prevent the causes of respiratory disease in Thailand. We will be randomly selecting 3,500 residents 65 years or older from That Phanom and Plapak districts.

You are invited to join this study because you are 65 years or older. You were selected at random from your sub-district. You are free to join the study or not. If you decide not to join, nothing bad will happen. If you agree to join, we will ask a few questions about your home life and health. The questionnaire should take about thirty minutes. You do not have to answer any question you don't want to answer. You can stop the questionnaire or withdraw from the study at any time without reason. The study will be 2 years long. We will ask you for your phone number and we will contact you once every week for 2 years. Every week we will ask you if you have had any new cough or worsening of cough the previous week and also ask you to answer questions

regarding your health. The weekly questionnaire should take no more than 15 minutes. If you get sick with a new cough, or if you have a chronic cough that worsens, we will ask you to take nasal swabs for us. We will show you the correct way to take this swab. After you take the swab, we will ask you to store it in a tube in the refrigerator and to call us to pick up the nasal swab. After you take a nasal swab, we will ask you some questions about your experience. After you feel better, we will also ask you to answer some questions about the cost of treatment. We will ask you for permission to check your medical records to record the cost of being treated for a respiratory illness, and if you have COPD, to check how many times you visited the district hospital because of a COPD exacerbation. Each time you visit your sub-district health center or district hospital because of a respiratory illness, we will have a nurse take a nasal throat swab.

All the information we collect is confidential. All of the information will be kept under lock or password. No one but study staff can look at your information. Two years after the study results are made public, all records will be destroyed. The risks for joining this study are minimal. If you follow our instructions for how to swab your nose, you should have no side effects, although you may experience some discomfort from taking the nasal swab. There are no real benefits to you for participating. You will help us improve our influenza prevention program. You will initially receive 300 baht upon enrolling in the study. You will receive another 300 baht after 6 months and 400 baht at the end of the study at 12 months. You will be reimbursed the same way for the second year for a total of 2000 baht over the two years. This money is to reimburse you for your time and phone calls. If you need to go to the local health center or district hospital for a visit related to this study, you will also be compensated 100 baht for transportation.

Researcher has guaranteed that if you are injured as a result of being in this study, treatment will be available without any costs, and compensation for revenue losses during treatment will be provided, including other compensation for possible disability. For further information about this, you may contact Dr. Kriengkrai Prasert and research team at Nakhon Phanom Public Health Office.

If you have questions about your rights as a study participant, please contact the office of Secretary, Ethic Review Committee for Research in Human Subjects, Ministry of Public Health,

Department of Disease Control and Prevention, Ministry of Public Health, Tiwanon road, Nonthaburi 11000 Tel. 02-590-3149.

- -

2b. Participant consent form for Vaccine Effectiveness Study

Study Title: Flu 8: Measuring the effectiveness of the trivalent inactivated influenza vaccine in persons aged 65 years and older in Thailand

Date of Consent Date............Month................................Year............................

Before signing this form, I received clear explanation from the health volunteer on study objectives, methods, risk or adverse event, and benefits from the study. I clearly understood everything.

The health volunteer answered all of my questions and I am satisfied with that.

I am willing to participate in this study. I can stop and withdraw from the study anytime. My action on participating in or withdrawing from the study will not affect my right to medical care.

The health volunteer guarantees that the study team will keep my personal data secret and will only disclose in a form of study summary or disclose to people who are related to study support or monitor this study.

The health volunteer has guaranteed that if I am injured as a result of being in this study, treatment will be available without any costs, and compensation for revenue losses during treatment will be provided, including other compensation for possible disability. For further information about this, I may contact Dr. Kriengkrai Prasert and research team at Nakhon Phanom Public Health Office.

I carefully read study details and this consent form. I received all explanations and clearly understand. I am willing to sign the form.

Name..Participant

Name..Researcher

Name..Witness

* I am an illiterate person. The researcher read study details and this consent form for me. I received all explanations and clearly understand. I am willing to give a finger print into this form.

Finger print...Participant

Name...Researcher

Name...Witness

2c. Participant information sheet for Immunogenicity (Objective 3)

Flesch-Kincaid Grade Level: 6.9

Research name: Flu 8: Measuring the effectiveness of the trivalent inactivated influenza vaccine in persons aged 65 years and older in Thailand

Principal Investigator

Dr. Kriengkrai Prasert Position: Medical physician senior professional levelPlace of work: Nakhon Phanom Public Health Office

Tel. Office phone 042-511410 Mobile phone: 081-975-5460 Fax 042-512 463

Co-Principal Investigator (Provincial Health Office)

Dr. Preeda Worahan

Tel. Mobile phone: 088-563-7079 and 081-871-9863

We are doing a small study in Nakhon Phanom as part of a larger study to measure how well and how long the flu vaccine protects persons aged 65 years and older. We want to see how long the antibodies from the flu vaccine stay in the body. We also want to know how many antibodies are produced in the body from the flu vaccine. This information will help us learn when and how often the flu vaccine should be taken. We plan to enroll a total of 384 participants from Plapak and That Phanom District Hospitals.

You are invited to join this study because you are 65 years or older and intend to take the flu vaccine, but have not yet received it in the year 2015. You are free to join the study or not. If you decide not to join, nothing bad will happen. This part of the study is 1 year long. If you agree to join, we will ask you to draw 5cc of blood four times, once right before you get the flu vaccine today, and then 28, 180 and 360 days after you get the flu vaccine. We will test this blood to see how many antibodies against flu you have at the time you gave us blood. We will schedule the appointments for you at the district hospital. If you are unable to go to the district hospital, we will send a research nurse to come to your home to draw the blood. You can stop and leave this study at any time without reason. We will ask you for your phone number and we will remind

you of your scheduled appointment. Getting your blood drawn should take no more than 15 minutes.

All the information we collect is confidential. All of the information will be kept under lock or password. No one but study staff can look at the results of your blood test. Two years after the study results are made public, all records will be destroyed. The risks for joining this study are minimal and may include experiencing discomfort, mild redness, or soreness at the local site of the blood draw. Trained nurses will withdraw the blood. There are no real benefits to you for participating. You will help us improve our influenza prevention program. You will receive 300 baht for each blood draw.

Researcher has guaranteed that if you are injured as a result of being in this study, treatment will be available without any costs, and compensation for revenue losses during treatment will be provided, including other compensation for possible disability. For further information about this, you may contact Dr. Kriengkrai Prasert and research team at Nakhon Phanom Public Health Office.

If you have questions about your rights as a study participant, please contact the office of Secretary, Ethic Review Committee for Research in Human Subjects, Ministry of Public Health, Department of Disease Control and Prevention, Ministry of Public Health, Tiwanon road, Nonthaburi 11000 Tel. 02-590-3149.

- -

2d. Consent form for Immunogenicity study

Study Title: Flu 8: Measuring the effectiveness of the trivalent inactivated influenza vaccine in persons aged 65 years and older in Thailand

Date of Consent Date............Month................................Year............................

Before signing this form, I received clear explanation from the health volunteer on study objectives, methods, risk or adverse event, and benefits from the study. I clearly understood everything.

The study team answered to all of my questions and I am satisfied with that.

I am willing to participate in this study. I can stop and withdraw from the study anytime. My action on participating in or withdrawing from the study will not affect my right to medical care.

The study team guarantees that the study team will keep my personal data secret and will only disclose in a form of study summary or disclose to people who are related to study support or monitor this study.

The study team has guaranteed that if I am injured as a result of being in this study, treatment will be available without any costs, and compensation for revenue losses during treatment will be provided, including other compensation for possible disability. For further information about this, I may contact Dr. Kriengkrai Prasert and research team at Nakhon Phanom Public Health Office.

I carefully read study details and this consent form. I received all explanations and clearly understand. I am willing to sign the form.

Name...Participant

Name...Researcher

Name...Witness

* I am an illiterate person. The study team read study details and this consent form for me. I received all explanations and clearly understand. I am willing to give a finger print into this form.

Finger print..Participant

Name... ....Researcher

Name..Witness

2e. Blood draw appointment sheet (Objective 2.2)

Name/ID___________________________________ Phone # _______________________

I need to go to ____ on:

(District Hospital)

- ☐ Day 0 _______________________________________

  (Day/Month/Year)

  - To have 5 cc blood drawn (before receiving flu vaccine)
  - Receive flu vaccine
- ☐ Day 28 (1 month)____________________________________

  (Day/Month/Year)

  - To have 5 cc blood drawn
- ☐ Day 180 (6 months)____________________________________

  (Day/Month/Year)

  - To have 5 cc blood drawn
- ☐ Day 360 (12 months)____________________________________

  (Day/Month/Year)

  - To have 5 cc blood drawn

If you need assistance to get to the hospital or you need to reschedule your appointment, please call the study team at ______________________ (Phone number)

Additional Notes:

Please circle appointment days below for participant:

มิถุนายน 2015

วัน จันทร์	วัน อังคาร	วัน พุธ	วัน พฤหัสบดี	วัน ศุกร์	วัน เสาร์	วัน อาทิตย์
1	2	3	4	5	6	7
8	9	10	11	12	13	14
15	16	17	18	19	20	21
22	23	24	25	26	27	28
29	30					

กรกฎาคม 2015

วัน จันทร์	วัน อังคาร	วัน พุธ	วัน พฤหัสบดี	วัน ศุกร์	วัน เสาร์	วัน อาทิตย์
		1	2	3	4	5
6	7	8	9	10	11	12
13	14	15	16	17	18	19
20	21	22	23	24	25	26
27	28	29	30	31		

สิงหาคม 2015

วัน จันทร์	วัน อังคาร	วัน พุธ	วัน พฤหัสบดี	วัน ศุกร์	วัน เสาร์	วัน อาทิตย์
					1	2
3	4	5	6	7	8	9
10	11	12	13	14	15	16
17	18	19	20	21	22	23
24	25	26	27	28	29	30
31						

กันยายน 2015

วัน จันทร์	วัน อังคาร	วัน พุธ	วัน พฤหัสบดี	วัน ศุกร์	วัน เสาร์	วัน อาทิตย์
	1	2	3	4	5	6
7	8	9	10	11	12	13
14	15	16	17	18	19	20
21	22	23	24	25	26	27
28	29	30				

ตุลาคม 2015

วัน จันทร์	วัน อังคาร	วัน พุธ	วัน พฤหัสบดี	วัน ศุกร์	วัน เสาร์	วัน อาทิตย์
			1	2	3	4
5	6	7	8	9	10	11
12	13	14	15	16	17	18
19	20	21	22	23	24	25
26	27	28	29	30	31	

พฤศจิกายน 2015

วัน จันทร์	วัน อังคาร	วัน พุธ	วัน พฤหัสบดี	วัน ศุกร์	วัน เสาร์	วัน อาทิตย์
						1
2	3	4	5	6	7	8
9	10	11	12	13	14	15
16	17	18	19	20	21	22
23	24	25	26	27	28	29
30						

ธันวาคม 2015

วัน จันทร์	วัน อังคาร	วัน พุธ	วัน พฤหัสบดี	วัน ศุกร์	วัน เสาร์	วัน อาทิตย์
	1	2	3	4	5	6
7	8	9	10	11	12	13
14	15	16	17	18	19	20
21	22	23	24	25	26	27
28	29	30	31			

มกราคม 2016

วัน จันทร์	วัน อังคาร	วัน พุธ	วัน พฤหัสบดี	วัน ศุกร์	วัน เสาร์	วัน อาทิตย์
				1	2	3
4	5	6	7	8	9	10
11	12	13	14	15	16	17
18	19	20	21	22	23	24
25	26	27	28	29	30	31

กุมภาพันธ์ 2016

วัน จันทร์	วัน อังคาร	วัน พุธ	วัน พฤหัสบดี	วัน ศุกร์	วัน เสาร์	วัน อาทิตย์
1	2	3	4	5	6	7
8	9	10	11	12	13	14
15	16	17	18	19	20	21
22	23	24	25	26	27	28
29						

มีนาคม 2016

วัน จันทร์	วัน อังคาร	วัน พุธ	วัน พฤหัสบดี	วัน ศุกร์	วัน เสาร์	วัน อาทิตย์
	1	2	3	4	5	6
7	8	9	10	11	12	13
14	15	16	17	18	19	20
21	22	23	24	25	26	27
28	29	30	31			

เมษายน 2016

วัน จันทร์	วัน อังคาร	วัน พุธ	วัน พฤหัสบดี	วัน ศุกร์	วัน เสาร์	วัน อาทิตย์
				1	2	3
4	5	6	7	8	9	10
11	12	13	14	15	16	17
18	19	20	21	22	23	24
25	26	27	28	29	30	

พฤษภาคม 2016

วัน จันทร์	วัน อังคาร	วัน พุธ	วัน พฤหัสบดี	วัน ศุกร์	วัน เสาร์	วัน อาทิตย์
						1
2	3	4	5	6	7	8
9	10	11	12	13	14	15
16	17	18	19	20	21	22
23	24	25	26	27	28	29
30	31					

มิถุนายน 2016

วัน จันทร์	วัน อังคาร	วัน พุธ	วัน พฤหัสบดี	วัน ศุกร์	วัน เสาร์	วัน อาทิตย์
		1	2	3	4	5
6	7	8	9	10	11	12
13	14	15	16	17	18	19
20	21	22	23	24	25	26
27	28	29	30			

กรกฎาคม 2016

วัน จันทร์	วัน อังคาร	วัน พุธ	วัน พฤหัสบดี	วัน ศุกร์	วัน เสาร์	วัน อาทิตย์
				1	2	3
4	5	6	7	8	9	10
11	12	13	14	15	16	17
18	19	20	21	22	23	24
25	26	27	28	29	30	31

สิงหาคม 2016

วัน จันทร์	วัน อังคาร	วัน พุธ	วัน พฤหัสบดี	วัน ศุกร์	วัน เสาร์	วัน อาทิตย์
1	2	3	4	5	6	7
8	9	10	11	12	13	14
15	16	17	18	19	20	21
22	23	24	25	26	27	28
29	30	31				

2f. Participant information sheet for Additional Immunogenicity Year 2 study (Objective 3)

Flesch-Kincaid Grade Level: 6.9

Research name: Flu 8: Measuring the effectiveness of the trivalent inactivated influenza vaccine in persons aged 65 years and older in Thailand

Principal Investigator

Dr. Kriengkrai Prasert Position: Medical physician senior professional level

Place of work: Nakhon Phanom Public Health Office

Tel. Office phone 042-511410 Mobile phone: 081-975-5460 Fax 042-512 463

Co-Principal Investigator (Provincial Health Office)

Dr. Preeda Worahan

Tel. Mobile phone: 088-563-7079 and 081-871-9863

You are enrolled in a sub-study as a part of a larger study to measure how well and how long the flu vaccine protects persons aged 65 years and older. Your blood has been drawn three times prior to this day (before vaccination, 28 days after vaccination and180 days after vaccination) and are scheduled to have another blood draw today (360 days after first vaccination).

Scientific information suggest that previous flu vaccination may decrease immune response level. To get a confirmative evidence which will provide benefit to the elderly, we have extended the study in which, your blood will be drawn one more time, 28 days after the next vaccination. In this study, the volunteers will already have flu vaccine at least one time (last year) and another time this year, that permits comparison of immune response levels of these vaccinations.

You are invited to join this study if you intend to take the flu vaccine, and have not yet received it in the year 2016. You are free to join the study or not. If you decide not to join, nothing bad will happen. This part of the study is an extension of 1 month from this day. If you agree to join, we will ask you to draw 5cc of blood one time, 28 days after getting vaccine today. We will test this blood to see how many antibodies against flu you have at the time you gave us

blood. We will schedule the appointments for you at the district hospital. If you are unable to go to the district hospital, we will send a research nurse to come to your home to draw the blood. You can stop and leave this study at any time without reason. We will ask you for your phone number and we will remind you of your scheduled appointment. Getting your blood drawn should take no more than 15 minutes.

All the information we collect is confidential. All of the information will be kept under lock or password. No one but study staff can look at the results of your blood test. Two years after the study results are made public, all records will be destroyed. The risks for joining this study are minimal and may include experiencing discomfort, mild redness, or soreness at the local site of the blood draw. Trained nurses will withdraw the blood. There are no real benefits to you for participating. You will help us improve our influenza prevention program. You will receive 300 baht for each blood draw.

Researcher has guaranteed that if you are injured as a result of being in this study, treatment will be available without any costs, and compensation for revenue losses during treatment will be provided, including other compensation for possible disability. For further information about this, you may contact Dr. Kriengkrai Prasert and research team at Nakhon Phanom Public Health Office.

If you have questions about your rights as a study participant, please contact the office of Secretary, Ethic Review Committee for Research in Human Subjects, Ministry of Public Health, Department of Disease Control and Prevention, Ministry of Public Health, Tiwanon road, Nonthaburi 11000 Tel. 02-590-3149.

- -

2g. Consent form for Additional Immunogenicity study Year 2

Study Title: Flu 8: Measuring the effectiveness of the trivalent inactivated influenza vaccine in persons aged 65 years and older in Thailand

Date of Consent Date............Month................................Year............................

Before signing this form, I received clear explanation from the health volunteer on study objectives, methods, risk or adverse event, and benefits from the study. I clearly understood everything.

The study team answered to all of my questions and I am satisfied with that.

I am willing to participate in this study. I can stop and withdraw from the study anytime. My action on participating in or withdrawing from the study will not affect my right to medical care.

The study team guarantees that the study team will keep my personal data secret and will only disclose in a form of study summary or disclose to people who are related to study support or monitor this study.

The study team has guaranteed that if I am injured as a result of being in this study, treatment will be available without any costs, and compensation for revenue losses during treatment will be provided, including other compensation for possible disability. For further information about this, I may contact Dr. Kriengkrai Prasert and research team at Nakhon Phanom Public Health Office.

I carefully read study details and this consent form. I received all explanations and clearly understand. I am willing to sign the form.

Name...Participant

Name...Researcher

Name...Witness

* I am an illiterate person. The study team read study details and this consent form for me. I received all explanations and clearly understand. I am willing to give a finger print into this form.

Finger print...Participant

Name.. ....Researcher

Name..Witness

3a. Village leader information sheet for Vaccine Effectiveness Study (including immunogenicity)

Flesch-Kincaid Grade Level: 6.9

Research name: Flu 8: Measuring the effectiveness of the trivalent inactivated influenza vaccine in persons aged 65 years and older in Thailand

Principal Investigator

Dr. Kriengkrai Prasert Position: Medical physician senior professional level

Place of work: Nakhon Phanom Public Health Office

Tel. Office phone: 042-511 410 Mobile phone: 081-975-5460 Fax 042-512 463

Co-Principal Investigator (Provincial Health Office)

Dr. Preeda Worahan

Tel. Mobile phone: 088-563 7079 and 081-871-9863

Hello. My name is ______________________________. I work for the Ministry of Public Health and the Provincial Health Office. We are doing a large, 2 year study in Nakhon Phanom. The purpose of this study is to learn how well the flu vaccine works in Thailand to stop people from becoming sick and to save money on treatment. We also want to know how having an acute respiratory infection effects health. We also want to know how often people get sick from the respiratory syncytial virus. This information will help us learn how to prevent the causes of respiratory disease in Thailand.

Your village has randomly been selected to join this study. You are free to let your village join the study or not. If you decide not to join, nothing bad will happen. If you agree, we will interview some randomly selected elderly and spend approximately 15 minutes to introduce the study to them. We will take baseline questionnaires from them regarding their health. We will contact them every week for the next 2 years to see if they have become ill and monitor their health. We will show them how to swab their noses if they get a respiratory illness. We will test

the swabs to see if they are sick due to influenza or the respiratory syncytial virus. After they feel better from their respiratory illness, we will ask them about their costs of being sick and treatment. A smaller number of participants who go to the District Hospital to have the flu vaccine will also be asked to have 5cc of their blood drawn four times over 1 year. We will test this blood to see how well and how long the flu vaccine protects the elderly from the flu. We will delete all personal identifying information after we clean the data. All the information we collect is confidential. All of the information will be kept under lock. No one but study staff can look at their information. Two years after the study results are made public, all records will be destroyed. There are minimal risks for joining this study including discomfort from respiratory swabbing and redness and soreness from having blood drawn. There are also no real benefits. The community will help us improve our influenza prevention program.

If you have questions about your rights or the rights of the study participants, please contact the office of Secretary, Ethic Review Committee for Research in Human Subjects, Ministry of Public Health,

Department of Disease Control and Prevention, Ministry of Public Health, Tiwanon road, Nonthaburi 11000 Tel. 02-590-3149.

- -

3b. Village leader consent form for Vaccine Effectiveness Study including Immunogenicity

Study Title: Flu 8: Measuring the effectiveness of the trivalent inactivated influenza vaccine in persons aged 65 years and older in Thailand

Date of Consent Date............Month.................................Year............................

Before signing this form, I received clear explanation from the researcher on study objectives, methods, risk or adverse event, and benefits from the study. I clearly understood everything.

The researcher answered to all of my questions and I am satisfied with that.

I am willing to allow the elderly in my village to participate in this study. We can stop and withdraw from the study anytime. Our actions on participating in or withdrawing from the study will not affect our right to medical care.

The researcher guarantees to keep our personal data secret and will only disclose in a form of study summary or disclose to people who are related to study support or monitor this study.

I carefully read study details and this consent form. I received all explanations and clearly understand. I am willing to sign the form.

Name..Village Leader

Name...Researcher

3a. Personal Identifying Information, Eligibility and Consent (For Health Volunteers)

Name of selected participant		
Address		Village/Sub-district
Name of health volunteer		
Date and time of first attempted visit		Outcome:
Date and time of second attempted visit		Outcome
Date and time of third attempted visit		Outcome

Outcome codes:

1. Interview completed
2. Interview partially completed – will return to finish
3. Interview partially completed – unable to return to finish
4. Refused
5. Ineligible
6. Absent and unlikely to return in next 7 days
7. Absent temporarily – will try again
8. Moved within study area – will try to contact
9. Moved outside of study area – cannot contact
10. Died. Provide date of death
11. No one knows the participant/Cannot be located

Describe how to find the house		
Home phone number		
Cell phone number		
Is there a caregiver?	1 Yes<br>2 No	Caregiver's name:
Phone number for caregiver?		Circle the number that we should use for the weekly call.
Participant's age (years)		
Have you lived in Nakhon Phanom since Songkran 2014?	1 Yes<br>2 No	

Screening form for influenza vaccine coverage and vaccination determinant for elderly age 65 years and over

Documentation of eligibility

No.	Questions and filters (variable names)	Coding categories	*Skip*
1	What is your date of birth?	__/__/__ (dd/mm/yy)<br>77 Incomplete date<br>99 Declined to answer	*If complete date, skip to 3*

If month and day are unknown, use 15 May. If year is unknown, ask for age and calculate year of birth. If year and month is known, but date is not, use the 15th of the month.			
2	Calculated date of birth?	__/__/__ (dd/mm/yy)	
3	Was participant born on or before 1 May, 1950 (or estimated to be on or before this date)?	1 Yes<br>2 Yes: DOB cannot be determined but is clearly eligible<br>3 Not eligible	
4	Where did you spend most of your time since Songkran 2014?	1 Within this district<br>2 Outside of this district but within Nakhon Phanom Province<br>3 Outside of Nakhon Phanom Province<br>77 Do not know	
5	Has the participant been living in this district since Songkran 2014?	1 Yes<br>2 No	
If yes to both questions 3 and 5,participant is still eligible; please continue with eligibility questions.<br>*If not eligible, record Outcome=5 for this visit, above, and thank the participant for his or her time.*			

6	Do you have any of the following conditions?<br>• body disabilities that may impact ability to do a nasal self-swab or telephone interview (i.e. elderly with stroke)<br>• history of tumor in the nose or nearby areas<br>• history of a bleeding disorder (i.e. Hemophilia)<br>• get nosebleeds often<br>• any illness or acute medical condition that could prevent your full participation	1 Yes<br>2 No<br>1 Yes<br>2 No<br>1 Yes<br>2 No<br>1 Yes<br>2 No<br>1 Yes<br>2 No	
If the elderly participant answers yes to any of the above questions, record Outcome=5 for this visit, above. Thank participant for their time and let them know that they do not qualify for the study. Otherwise, continue to document consent form.			

Documentation of consent for VE study

Participant has read and signed the informed consent form for VE study?	1 Yes<br>2 No
If no, please specify the reason for refusal below and Thank the participant for his or her time.<br>____	
7. Participant's 13 digit identification number:	
8.Study identification number:	

9.Latitude and longitude(Turn on the GPS in the electronic PDA outside or inside of the participant's household and write down the latitude and longitude it shows when the satellite finds the location of the PDA into the screening form on the PDA):
10. Which health center is closest to your home?
11. How far away is the closest health center from your home? km
Have study team member begin enrollment questionnaire.

4b. Enrollment Questionnaire (for Study Team)

No.	Questions and filters (variable names)	Coding categories	*Skip*
1	Study id number		
2	Date of interview		
3	Participant's age		
Now we would like to ask you some questions about how you are feeling and how well you are able to take care of yourself.			
4	In general, compared to other people your age, would you say that your health is poor, fair, good, very good or excellent?	1 Very poor<br>2 Bad<br>3 Fair<br>4 Good<br>5 Very good or excellent<br>77 Do not know<br>99 Declined to answer	
5	Have you experienced any severe illness in the last year?	1 Yes<br>2 No<br>77 Do not know<br>99 Declined to answer	
6	Did you have to be hospitalized for any illness in the last year?	1 Yes<br>2 No<br>77 Do not know<br>99 Declined to answer	

7	Did you fall since Sonkran Festival 2014?	1 Yes<br>2 No<br>77 Do not know<br>99 Declined to answer	*If no, do not know or declined to answer skip to 9*
8	If so, how many times did you fall since Sonkran Festival 2014?	________	
9	Please tell us what types of health insurance you have. *Listen to the answers and check all that are mentioned. When the participant has finished responding, prompt once for any additional effects.*	1 universal coverage<br>2 Government health insurance/state enterprise<br>3 Private health insurance<br>4 Fee treatment<br>5 Not eligible<br>77 Do not know<br>99 Declined to answer	

	How much difficulty, <u>on average</u>, do you have with the following physical activities:						
	**Activity**	**No difficulty**	**A little difficulty**	**Some difficulty**	**A lot of difficulty**	**Unable to do**	**Unable to answer**
10	Stooping, crouching or kneeling	☐	☐	☐	☐	☐	☐

11	Lifting or carrying objects as heavy as 5 kilos?	☐	☐	☐	☐	☐	☐
12	Reaching or extending arms above shoulder level?	☐	☐	☐	☐	☐	☐
13	Writing or handling and grasping small objects?	☐	☐	☐	☐	☐	☐
14	Walking a half of a kilometer	☐	☐	☐	☐	☐	☐
15	Heavy housework such as scrubbing floors or washing windows	☐	☐	☐	☐	☐	☐

16	Because of your health or a physical condition, do you have any difficulty shopping for personal items (like medications or toilet items)?	1 Yes<br>2 No<br>3 Don't do<br>99 Declined to answer	*If no or declines to answer, skip to 19*<br>*If 'don't do,' skip to 18*
17	If yes, do you get help with shopping?	1 Yes<br>2 No<br>99 Declined to answer	*Skip to 19*
18	If you don't shop, is that because of your health?	1 Yes<br>2 No<br>99 Declined to answer	

19	Because of your health or a physical condition, do you have any difficulty managing money (like keeping track of expenses or paying bills)?	1 Yes<br>2 No<br>3 Don't do<br>99 Declined to answer	*If no or declines to answer, skip to 22*<br>*If 'don't do,' skip to 21*
20	If yes, do you get help with managing money?	1 Yes<br>2 No<br>99 Declined to answer	*Skip to 22*
21	If you don't manage your money, is that because<br>of your health?	1 Yes<br>2 No<br>99 Declined to answer	
22	Because of your health or a physical condition, do you have any difficulty walking across the room?	1 Yes<br>2 No<br>3 Don't do<br>99 Declined to answer	*If no or declines to answer, skip to 25*<br>*If 'don't do,' skip to 24*
23	If yes, do you get help with walking?	1 Yes<br>2 No	*Skip to 25*

		99 Declined to answer	
24	If you can't walk across the room, is that because of your health?	1 Yes<br>2 No<br>99 Declined to answer	
25	Because of your health or a physical condition, do you have any difficulty doing light housework (like washing dishes, straightening up or light cleaning)?	1 Yes<br>2 No<br>3 Don't do<br>99 Declined to answer	*If no or declines to answer, skip to 28*<br>*If 'don't do,' skip to 27*
26	If yes, do you get help with light housework?	1 Yes<br>2 No<br>99 Declined to answer	*Skip to 28*
27	If you can't do light housework, is that because of your health?	1 Yes<br>2 No<br>99 Declined to answer	
28	Because of your health or a physical condition, do you have any difficulty bathing or showering?	1 Yes<br>2 No<br>3 Don't do<br>99 Declined to answer	*If no or declines to answer, skip to 31*

			If 'don't do,' skip to 30
29	If yes, do you get help with bathing or showering?	1 Yes<br>2 No<br>99 Declined to answer	*Skip to 31*
30	If you can't bathe or shower, is that because of<br>your health?	1 Yes<br>2 No<br>99 Declined to answer	
31	How often do you leave your house for any reason (work, errands, exercise or to visit friends and family, etc.)?	0 Rarely or never<br>1 About once a month<br>2 A few times a week<br>3 Every day<br>77 Do not know<br>99 Declined to answer	
32	How would you rate your memory?	0 Significant memory loss, confusion or dementia<br>1 Moderate memory loss, confusion or dementia<br>2 Minor memory loss<br>3 No memory problems<br>77 Do not know<br>99 Declined to answer	

Now I would like to ask you about your health history. Please tell me whether you have been diagnosed by a physician with any of the following conditions:			
33	Chronic lung disease	1 Yes<br>2 No<br>77 Do not know<br>99 Declined to answer	*If no, skip to 35*
34	If yes, please specify:	1 COPD<br>2 Emphysema<br>3 Asthma<br>4 Fibrosis<br>5 Chronic bronchitis<br>6 Tuberculosis<br>7 Lung cancer<br>8 Other<br>Specify:__________<br>77 Do not know<br>99 Declined to answer	
35	Chronic heart and circulatory disease	1 Yes<br>2 No<br>77 Do not know<br>99 Declined to answer	*If no, skip to 37*
36	If yes, please specify:	1 Hypertension<br>2 Cardiomyopathy<br>3 Coronary artery disease	

		4 Heart valve disease<br>5 Abnormal heart rhythms or arrhythmias<br>6 Other<br>77 Do not know<br>99 Declined to answer	
37	Cerebrovascular disease (stroke)	1 Yes<br>2 No<br>77 Do not know<br>99 Declined to answer	
38	Chronic kidney disease	1 Yes<br>2 No<br>77 Do not know<br>99 Declined to answer	
39	Chronic liver disease	1 Yes<br>2 No<br>77 Do not know<br>99 Declined to answer	
40	Neurologic/neuromuscular disorder (including muscular dystrophy, cerebral palsy)	1 Yes<br>2 No<br>77 Do not know<br>99 Declined to answer	

41	Hemoglobinopathy, including thalassemia	1 Yes<br>2 No<br>77 Do not know<br>99 Declined to answer	*If no, skip to 43*
42	If yes, please specify	1 Thalassemia<br>2 Other<br>77 Do not know<br>99 Declined to answer	
43	Metabolic disease, including diabetes	1 Yes<br>2 No<br>77 Do not know<br>99 Declined to answer	*If no, skip to 45*
44	If yes, please specify	1 Diabetes<br>2 Other<br>77 Do not know<br>99 Declined to answer	
45	Do you have any immunosuppressive conditions (such as chemotherapy)	1 Yes<br>2 No<br>77 Do not know<br>99 Declined to answer	*If no, skip to 47*
46	If yes, please specify	1 HIV infection<br>2 Chemotherapy<br>3 Other<br>77 Do not know	

		99 Declined to answer	
47	Lupus	1 Yes<br>2 No<br>77 Do not know<br>99 Declined to answer	
48	Cancer, not reported above	1 Yes<br>2 No<br>77 Do not know<br>99 Declined to answer	
49	Do you have a history of smoking ≥ 1 cigarette/day for at least 1 year	1 Yes<br>2 No<br>77 Do not know<br>99 Declined to answer	*If no, skip to question 54*
50	Are you currently smoking?	1 Yes<br>2 No<br>77 Do not know<br>99 Declined to answer	*If yes, answer question 51 and 52, but skip question 53*
51	How many cigarettes on average do you or did you smoke per day?	_________ cigarettes (1 pack has 14 cigarettes)<br>77 Do not know<br>99 Declined to answer	

52	When did you start smoking?	_____________(MM/YY)<br><br>77 Do not know<br><br>99 Declined to answer	
53	When did you quit smoking?	_____________<br><br>(MM/YY)	
We are almost finished with the questionnaire. We would like to ask you a few questions about your economic and personal situation.			
54	Sex	1 Male<br><br>2 Female	
55	Are you currently married?	1 Married<br><br>2 Widowed<br><br>3 Divorced/separated<br><br>4 Single (never married)<br><br>77 Do not know<br><br>99 Declined to answer	
56	How many other people live with you in this house?	______________________<br><br>77 Incomplete data<br><br>99 Declined to answer	
57	If you become ill, where do you normally seek care? *Check only one answer.*	1 District hospital<br><br>Specify:__________<br><br>2 Provincial hospital<br><br>Specify: __________	

		3 Sub-district health center<br>Specify: ___________<br>4 Private physician<br>5 Don't seek care/drug store<br>6 Other<br>77 Do not know<br>99 Declined to answer	
58	What is the highest level of education you completed?	0 Never attended school<br>1 Some primary<br>2 Completed primary<br>3 Some secondary<br>4 Completed secondary<br>5 Some post-secondary education<br>6 Graduate degree<br>77 Do not know<br>99 Declined to answer	
59	Are you the head of your household?	1 Yes<br>2 No<br>77 Do not know<br>99 Declined to answer	*If yes, skip to 61*
60	If no, what is the highest level of education completed by the head of household?	0 Never attended school	

		1 Some primary<br>2 Completed primary<br>3 Some secondary<br>4 Completed secondary<br>5 Some post-secondary education<br>6 Graduate degree<br>77 Do not know<br>99 Declined to answer	
61	What is your average monthly household income in Thai baht?	1 Less than 5,000<br>2 5,000-9,999<br>3 10,000-19,999<br>4 20,000-29,999<br>5 30,000-39,999<br>6 40,000 or more<br>77 Do not know<br>99 Declined to answer	
Please tell us whether your (or your family member's) household has any of the following items:			
62	Motorcycle, tuk tuk, etc.	1 Yes 2 No 77 DK<br>99 Decl	
63	Motor vehicle (car or pickup truck)	1 Yes 2 No 77 DK<br>99 Decl	
64	Radio	1 Yes 2 No 77 DK<br>99 Decl	*If no, skip to 66*

65	Have you listened to it?	1 Yes 2 No 77 DK<br>99 Decl	
66	Television	1 Yes 2 No 77 DK<br>99 Decl	*If no, skip to 68*
67	have you watched it ?	1 Yes 2 No 77 DK<br>99 Decl	
68	Refrigerator	1 Yes 2 No 77 DK<br>99 Decl	
69	Computer	1 Yes 2 No 77 DK<br>99 Decl	
70	Washing machine	1 Yes 2 No 77 DK<br>99 Decl	
71	Jewelry	1 Yes 2 No 77 DK<br>99 Decl	
72	Telephone (mobile or landline)	1 Yes 2 No 77 DK<br>99 Decl	
73	If you said you had a telephone, is it a mobile or landline?	1 Mobile<br>2 Landline<br>77 Not sure<br>99 Declined to answer	
74	Do you make and receive calls on the phone?	1 Makes and receives calls<br>2 Receives calls only<br>3 Neither makes nor receives calls	

		77 Do not know<br>99 Declined to answer	
75	*What kind of house does the participant live in?(Observe)*	1 House (formal)<br>2 Informal setting	
76	With what type of fuel do you normally use to cook, or someone else uses to cook for you? *Check only one answer.*	1 Electricity<br>2 Gas (propane, LPG)<br>3 Coal<br>4 Charcoal<br>5 Kerosene<br>6 Firewood or scrap wood<br>7 Other<br>77 Do not know<br>99 Declined to answer	
77	What is the main source of your drinking water? *Check only one answer.*	1 Piped water (government, private or communal)<br>2 Well/tube well<br>3 Bottled water<br>4 Pond, spring, river, lake<br>5 Other<br>77 Do not know<br>99 Declined to answer	
78	What type of toilet do you have? *Check only one answer.*	1 Flush toilet<br>2 Squat	

		3 Both flush and squat<br>4 Bucket toilet<br>5 None (outdoors)<br>6 Other<br>77 Do not know<br>99 Declined to answer	
79	Have you experienced any shortage of food in the last year?	1 Yes<br>2 No<br>77 Do not know<br>99 Declined to answer	
Thank you very much for your time and patience. We will now explain how to perform the nasal swab with all necessary materials.			

End of Questionnaire

4c. Weekly Contact Questionnaire (For Health Volunteer)

Study identification number	
Caller's name	
Type of Weekly contact	Please choose: In Person Phone
Week of follow-up	
Date	(DD/MM/YY)
Results	1 Declined (wishes to discontinue with the study)<br>Specify reason:<br>2 Unable to contact (this is the ____ consecutive time)<br>3 Contact made and interview completed
(On phone or in person) Hello, my name is _______ and I am a study team member from the Ministry of Public Health. May I please speak to ________(participant or caregiver of participant)?	
[If not a home] When would be a good time to call back to speak to him/her?<br>____	

No.	Questions and filters (variable names)	Coding categories	*Skip*
If the participant has been sick and took nasal self-swabs or had a nasopharyngeal swab taken recently, ask the following questions. Otherwise, skip to question 3.			
1	Have you recovered from your acute respiratory illness?	1 Yes<br>2 No<br>77 Do not know	

2	How many days ago did you recover from your acute respiratory illness?	1 Today<br>2 Yesterday<br>3 2 days ago<br>4 3 days ago<br>5 4 days ago<br>6 5 days ago<br>7 6 days ago<br>8 7 days ago	
Mark calendar/appointment sheet to call participant 1 week after they say they recovered from their respiratory illness to take the Functional Status Questionnaire and the Outcomes and Costs of ARI Questionnaire.			
3	In the last week, have you been sick with a new, acute illness?	1 Yes<br>2 No<br>77 Do not know	*If no, skip to end of question naire*
In the last week, have you had a NEW:			
4	Cough, or a worsening of a chronic cough?	1 Yes<br>2 No<br>77 Do not know	
5	Fever?	1 Yes<br>2 No<br>77 Do not know	
6	Nasal discharge/congestion?	1 Yes<br>2 No	

		77 Do not know	
7	Sore throat?	1 Yes<br>2 No<br>77 Do not know	
If no report of a new cough or worsening of a chronic cough, please thank patient for their time and let them know that they do not need to take nasal self-swabs this week. Go to end of survey.			
8	Did you already collect nasal swabs for the new illness?	1 Yes<br>2 No<br>77 Do not know	
If the participant reports a new cough or a worsening of a chronic cough and hasn't already taken a swab, schedule an appointment to collect nasal self- swabs as soon as possible. Fill out an ARI/SARI episode Questionnaire.			

END OF QUESTIONNAIRE

4d. ARI/SARI episode Questionnaire (For Health Volunteers)

Study identification number of caller	
Health Volunteer's name	
Date	(DD/MM/YY)
Thank you for notifying me.	

**No.**	**Questions and filters (variable names)**	**Coding categories**	***Skip***
1	In the last week, have you been sick with a new, acute illness?	1 Yes<br>2 No<br>77 Do not know	
In the last week, have you had a NEW:			
2	Cough, or a worsening of a chronic cough?	1 Yes<br>2 No<br>77 Do not know	
3	Fever?	1 Yes<br>2 No<br>77 Do not know	
4	Nasal discharge/congestion?	1 Yes<br>2 No<br>77 Do not know	
5	Sore throat?	1 Yes<br>2 No	

		77 Do not know	
If participant answered no for question 2, tell patient they do not need to take nasal swabs at this time and thank them. Go to end of Questionnaire.			
6	When did this illness begin?	1 Today<br>2 Yesterday<br>3 2 days ago<br>4 3 days ago<br>5 4 days ago<br>6 5 days ago<br>7 6 days ago<br>8 7 days ago	
7	At what time did the illness begin?	____________	
8	What day was the nasal self-swab taken	MM/DD/YY	
9	What time was the nasal self- swab taken	____________	
Collect a nasal self-swab and take to assigned health center.			

4e. ARI/SARI episode Questionnaire (For Trained Nurses in Health Centers)

Study identification number	
Nurse's name	
Date	
HN number	

Please check patient chart to see reason for admit and diagnosis and record below

____

Answer the questions below. Ask participant the following questions if not in medical record.

**No.**	**Questions and filters (variable names)**	**Coding categories**	***Skip***
1	Is the participant feeling sick with a new, acute illness?	1 Yes<br>2 No<br>77 Do not know	
Is the participant experiencing any of the following?			
2	Cough, or a worsening of a chronic cough?	1 Yes<br>2 No<br>77 Do not know	
3	Difficulty Breathing?	1 Yes<br>2 No<br>77 Do not know	
4	Fever?	1 Yes<br>2 No<br>77 Do not know	*If no or do not know, skip to question 6*

5	Is fever ≥38^0C	1 Yes<br>2 No<br>77 Do not know	
6	Nasal discharge/congestion?	1 Yes<br>2 No<br>77 Do not know	
7	Sore throat?	1 Yes<br>2 No<br>77 Do not know	
8	Is the patient hospitalized or does the patient need to be hospitalized?	1 Yes<br>2 No<br>77 Do not know	
9	When did this illness begin?	1 Today<br>2 Yesterday<br>3 2 days ago<br>4 3 days ago<br>5 4 days ago<br>6 5 days ago<br>7 6 days ago<br>8 7 days ago<br>9 More than 7 days ago	
If answer is no for question 2 and 3, do not take a nasopharyngeal swab and thank participant for their time. Otherwise, take a nasopharyngeal swab as directed in the Standard Operating Procedure.			
Call the study team to inform them about the participant's clinical visit.			

4f. Functional Status Questionnaire (For Health Volunteer)

Study identification number	
Name of Health volunteer (interviewer)	
Date	(DD/MM/YY)
Type of Weekly contact	Please choose: In Person Phone

We would like to ask you some questions about how you are feeling and how well you are able to take care of yourself.			
1	In general, compared to other people your age, would you say that your health is poor, fair, good, very good or excellent?	1 Very poor<br>2 Bad<br>3 Fair<br>4 Good<br>5 Very good<br>77 Do not know<br>99 Declined to answer	
Since the beginning of this study, or the last time we asked you these questions when you had a respiratory illness (whichever is most recent), have you			
2	experienced any severe illnesses?	1 Yes<br>2 No<br>77 Do not know<br>99 Declined to answer	
3	been hospitalized for any illness?	1 Yes<br>2 No	

		77 Do not know<br>99 Declined to answer	
4	fallen?	1 Yes<br>2 No<br>77 Do not know<br>99 Declined to answer	*If no, do not know or declined to answer skip to 6*
5	If so, how many additional times did you fall?	________	

How much difficulty, on average, do you have with the following physical activities:

	**Activity**	**No difficulty**	**A little difficulty**	**Some difficulty**	**A lot of difficulty**	**Unable to do**	**Unable to answer**
6	Stooping, crouching or kneeling	☐	☐	☐	☐	☐	☐
7	Lifting or carrying objects as heavy as 5 kilos?	☐	☐	☐	☐	☐	☐
8	Reaching or extending arms above shoulder level?	☐	☐	☐	☐	☐	☐
9	Writing or handling and grasping small objects?	☐	☐	☐	☐	☐	☐
10	Walking a half of a kilometer	☐	☐	☐	☐	☐	☐

11	Heavy housework such as scrubbing floors or washing windows	☐	☐	☐	☐	☐	☐

12	Because of your health or a physical condition, do you have any difficulty shopping for personal items (like medications or toilet items)?	1 Yes<br>2 No<br>3 Don't do<br>99 Declined to answer	*If no or declines to answer, skip to 15*<br>*If 'don't do,' skip to 14*
13	If yes, do you get help with shopping?	1 Yes<br>2 No<br>99 Declined to answer	*Skip to 15*
14	If you don't shop, is that because of your health?	1 Yes<br>2 No<br>99 Declined to answer	
15	Because of your health or a physical condition, do you have any difficulty managing money (like keeping track of expenses or paying bills)?	1 Yes<br>2 No<br>3 Don't do<br>99 Declined to answer	*If no or declines to answer, skip to 18*<br>*If 'don't do,' skip to 17*

16	If yes, do you get help with managing money?	1 Yes<br>2 No<br>99 Declined to answer	*Skip to 18*
17	If you don't manage your money, is that because<br>of your health?	1 Yes<br>2 No<br>99 Declined to answer	
18	Because of your health or a physical condition, do you have any difficulty walking across the room (using a cane or walker is okay)?	1 Yes<br>2 No<br>3 Don't do<br>99 Declined to answer	*If no or declines to answer, skip to 21*<br>*If 'don't do,' skip to 20*
19	If yes, do you get help with walking?	1 Yes<br>2 No<br>99 Declined to answer	*Skip to 21*
20	If you don't walk across the room, is that because<br>of your health?	1 Yes<br>2 No<br>99 Declined to answer	
21	Because of your health or a physical condition, do you have any difficulty doing light housework (like washing dishes, straightening up, or light cleaning)?	1 Yes<br>2 No<br>3 Don't do	*If no or declines to answer,*

		99 Declined to answer	*skip to 24*<br>*If 'don't do,' skip to 23*
22	If yes, do you get help with light housework?	1 Yes<br>2 No<br>99 Declined to answer	*Skip to 24*
23	If you don't do light housework, is that because of your health?	1 Yes<br>2 No<br>99 Declined to answer	
24	Because of your health or a physical condition, do you have any difficulty bathing or showering?	1 Yes<br>2 No<br>3 Don't do<br>99 Declined to answer	*If no or declines to answer, skip to 27*<br>*If 'don't do,' skip to 26*
25	If yes, do you get help with bathing or showering?	1 Yes<br>2 No<br>99 Declined to answer	*Skip to 27*
26	If you don't bathe or shower, is that because of your health?	1 Yes<br>2 No<br>99 Declined to answer	

27	How often do you leave your house for any reason (work, errands, exercise or to visit friends and family, etc.)?	0 Rarely or never<br>1 About once a month<br>2 A few times a week<br>3 Every day<br>77 Do not know<br>99 Declined to answer	
28	How would you rate your memory?	0 Significant memory loss, confusion or dementia<br>1 Moderate memory loss, confusion or dementia<br>2 Minor memory loss<br>3 No memory problems<br>77 Do not know<br>99 Declined to answer	
Please thank the participant for their time			

END OF QUESTIONNAIRE

4g. Outcomes and Costs of ARI/SARI Questionnaire (For Health Volunteer)

Study identification number	
Date	(DD/MM/YY)
HN number	
Type of Weekly contact	Please choose: In Person Phone

No.	Questions and filters (variable names)	Coding categories	*Skip*
1	Do you work? (for example, in the agricultural fields, run business, other job, etc.)	1 Yes<br>2 No<br>77 Do not know<br>99 Declined to answer	If answered No or Do not know, skip to question 3
2	How many days of work did you miss because of your illness?	_______ days	
3	Does your primary caregiver earn money in addition to taking care of you?	1 Yes<br>2 No<br>77 Do not know<br>99 Declined to answer	If No, skip to question 8
4	How many days was your caregiver unable to work because they were taking care of you?	______ days	
5	How much does the primary caretaker earn per day?	Average: ________Thai baht / day	

6	Did the primary caregiver lose any income by looking after you while you were sick?	1 Yes<br>2 No<br>77 Do not know<br>99 Declined to answer	If No, skip to question 9
7	How much?	________Thai baht	
8	Is there a risk of your caregiver losing their job for staying home to look after you?	1 Yes<br>2 No<br>77 Do not know<br>99 Declined to answer	
9	Did anyone else lose any income by looking after you?	1 Yes<br>2 No<br>77 Do not know<br>99 Declined to answer	If No, skip to question 14
10	How many days was the other household member who looked after you not able to work because of caring for you?	______ days	
11	How much does the other household member who looks after you earn per day?	Average: ________Thai baht / day	
12	Did the other household member lose any income by looking after you while you were sick?	1 Yes<br>2 No<br>77 Do not know<br>99 Declined to answer	If No, skip to question 14

13	How much?	________Thai baht	
14	Does your family need to give up purchasing something necessary in order to pay for treatment while you were sick? For example, food?	1 Yes<br>2 No<br>77 Do not know<br>99 Declined to answer	
15	How many days were there from the beginning of the illness until you recovered?	________ days	
16	During this whole illness, how many times did you see a medical doctor or a nurse for your illness? Please count all times until you recovered.	________times	
Please indicate the mechanisms you used to pay for the expenses caused by your illness?			
17	Government health plan	1 Yes<br>2 No	
18	Civil servant medical benefit scheme	1 Yes<br>2 No	
19	Health Insurance you purchased yourself (private insurance)	1 Yes<br>2 No	
20	Out of pocket	1 Yes<br>2 No	
21	Borrowed money	1 Yes<br>2 No	If No, skip to question 23
22	What was the interest rate?	____________%	

23	Other sources	1 Yes<br>2 No	If No, skip to question 25
24	Specify	______________________	
25	How much money did you spend on transportation fees to and from all visits to hospitals and clinics?	_____________Thai baht	
26	How much money did you spend on all medications not provided by the hospital or clinic (including herbals) and treatments (including traditional healers)	_____________Thai baht	
27	Which public health-centers/hospitals did you visit for your illness?	Please List:<br>1____________________<br>2____________________<br>3____________________<br>4____________________<br>5____________________	
28	Did you visit any private health centers during your illness?	1 Yes<br>2 No<br>77 Do Not Know<br>99 Declined to answer	
29	How much money did you spend on the total treatment of your illness at the private health centers? (Including all laboratory and	_____________Thai baht	

	examination tests, clinician fees, hospital fees, medication fees)		
30	How much money did your insurance spend on the total treatment of your illness at the private health centers? (including all laboratory and examination tests, clinician fees, hospital fees, medication fees)	_____________Thai baht	

___________END______________

4h. Screening and Enrollment Questionnaire for Immunogenicity study

Questionnaire 1: Personal Data to link in a separate database

Name of selected participant		
Participant Study ID Number		
Name of study team member		
District Hospital	Circle one: That Phanom Plapak	
Date (DD/MM/YY)		
Participant's Home phone number		
Participant's Cell phone number		
Is there a caregiver?	1 Yes<br>2 No	Caregiver's name:
Phone number for caregiver?		Circle the number that we should use for the weekly call.
Participant's age (years)		
Have you lived in Nakhon Phanom since Songkran 2014?	1 Yes<br>2 No	

Questionnaire 2: Screening form for influenza vaccine coverage and vaccination determinant for elderly age 65 years and over
Documentation of eligibility

Participant's Study ID number			
No.	Questions and filters (variable names)	Coding categories	*Skip*
1	What is your date of birth?	__/__/__ (dd/mm/yy)<br>77 Incomplete date<br>99 Declined to answer	*If complete date, skip to 3*
If month and day are unknown, use 15 May. If year is unknown, ask for age and calculate year of birth.			
2	Calculated date of birth?	__/__/__ (dd/mm/yy)	
3	Was participant born on or before 15 May, 1950 (or estimated to be on or after this date)?	1 Yes<br>2 Yes: DOB cannot be determined but is clearly eligible<br>3 Not eligible	
4	Where did you spend most of your time since Songkran 2014?	1 Within this district<br>2 Outside of this district but within Nakhon Phanom Province	

		3 Outside of Nakhon Phanom Province<br>77 Do not know	
5	Has the participant been living in this district since Songkran 2014?	1 Yes<br>2 No	
If yes to both questions 3 and 5, participant is still eligible; please continue with eligibility questions.<br>*If not eligible, thank the elderly for his or her time and let them know they are ineligible for the study.*			

6. Have you received the influenza vaccine in 2015?	1 Yes<br>2 No
If yes, end the questionnaire and thank elderly for his or her time and let them know they are ineligible for the study.	
7. Are you here to receive the influenza vaccine this year?	1 Yes<br>2 No<br>77 Do not know<br>99 Declined to Answer
If replied with no, do not know, or declined to answer, thank elderly for his or her time and let them know they are ineligible for the study<br>*If answered Yes, ask the following questions:*	
8. Are you allergic to egg, or do you have any known allergy or negative reaction to the flu vaccine?	1 Yes<br>2 No<br>3 Do not know

9. Do you have any medical condition that could prevent your full participation in having blood drawn?	1 Yes<br>2 No
If replied with yes to either question 8 or 9, thank elderly and let participant know they are not eligible for the immunogenicity part of the study. If answered No to both 8 and 9, please inform participant about immunogenicity study and ask for consent	

Documentation of consent for Immunogenicity study

Participant has read and signed the informed consent form for Immunogenicity study?	1 Yes<br>2 No
*If no, please specify the reason for refusal.*____<br>*If yes, ask the following baseline questions and record the following data*	
Participant's Identification Number (to obtain Influenza records)	
Participant's Study ID number	
Participant's HN number	
10. When, if ever, have you received the influenza vaccine in the past?	Please circle all that apply:<br>0 Never<br>1 Last year<br>2 2 to 4 years ago<br>3 5+ years ago<br>77 Do not know<br>99 Declined to answer

11. Do you have any conditions that make you immunocompromised (For example, chemotherapy, bone marrow or organ transplant, steroid medications, etc.)?	1 Yes<br>2 No<br>77 Do not Know<br>99 Declined to answer
Fill out blood titer appointment sheet with participant and record appointment dates for our records	1st blood draw/vaccine _______________________<br>2nd blood draw________________<br>3rd Blood draw________________<br>4th blood draw________________
Tell participant, "Thank you for your time and for enrolling in the immunogenicity study."<br>*Direct participant about where to go to have blood drawn prior to obtaining the influenza vaccine.*	

4i. Number and characteristic of people living with participant

1. How many people live with you now?

    1. None (stop answer here)
    2. One or more, please specify number ....person (keep answer question 2)

2. Please specify name, relationship, age and living time

Name	Sex	Age (year)*	Relationship**	Living time		If not living with you now, month and year when they moved out
				Living with you in May 2015?	If no, month and year when they moved in	
1						
2						
3						
4						
5						
6						
7						
8						
9						
10						

*specify in month if age less than 1 year

** 01 = wife or husband 02 = son or daughter 03 = brother or sister
04 = son-in-law or daughter-in-law 05 = grandchild 06 = parent
07 = other relative 08= friend 09= helper 10 = don't know

3. Is there anyone who lived with you for more than one month who is not listed in the table?

□ No □ Yes (please add detail to the table)

5a. Standard Operating Protocol for Nasal self-swab and Nasopharyngeal swab for Study Team and Nurses

Research nurses will teach participants to collect nasal swabs in the manner below and the written instructions will be provided to the elderly participant.

For the study team in the Community:

1. Show a video demonstrating the above technique to the participant step-by-step without the materials which follow the below written instructions "How to take a nasal self-swab"
2. Ask the participant to demonstrate the technique for taking a nasal self-swab without the materials to show that they understand the technique
3. Instruct the participant to take a nasal self-swab when they have a new onset of cough or worsening of chronic cough
4. Instruct the patient to store their nasal swab test tube in the refrigerator
5. Then instruct patient to immediately call their assigned health volunteer to schedule a time for the swab to be picked-up within 24 hours
6. Write health volunteer's telephone number for the patient on the written instruction sheet and let participant know that they can call the health volunteer if they have any concerns or need help.
7. Ask patient to verbally repeat steps 3 to 5 to demonstrate understanding

For nurses in the Health centers:

1. Fill out ARI/SARI episode Questionnaire (for Trained nurses in Health Centers), if participant meets criteria for a nasopharyngeal swab, proceed to next step.
2. Put on gloves and a mask
3. Take a nasopharyngeal swab according to written instructions "How to take a nasopharyngeal swab"
4. Place the labeled and filled test tube in the designated refrigerator provided at the health center

References for nasal self-swab and nasopharyngeal swab guidelines below:

1. Dhiman N, Miller RM, Finley JL, Sztajnkrycer MD, Nestler DM, Boggust AJ, Jenkins SM, Smith TF, Wilson JW, Cockerill FR, 3rd, Pritt BS: **Effectiveness of patient-collected swabs for influenza testing.** *Mayo Clin Proc* 2012, **87:**548-554.
2. **Specimen Collection.** (Centers for Disease Control and Prevention): National Health and Nutrition Examination Survey; 2000:1-47.
3. **WHO Guidelines for the collection of human specimens for laboratory diagnosis of avian influenza infection** [http://www.who.int/influenza/human_animal_interface/virology_laboratories_and_vaccines/guidelines_collection_h5n1_humans/en/]
4. **Nasopharyngeal swab specimen collection instructions: Influenza Testing**. West Virginia Department of Health and Human Resources [http://www.dhhr.wv.gov/oeps/disease/flu/documents/sentinel-providers/flu-collection-instructions.pdf]
5. **Nasopharyngeal swab collection for DFA; Fast Point**. Pathology Associates; The Clinical Laboratory Testing Manual. [http://webserver.pa-ucl.com/wwwdocs/cltm/Specimen%20Handling/Frame.htm]

How to take a nasal self-swab

Materials:

- Swab Labeled test tube with Transport Media (TM)
- Clean Glass or Cup
- Written Instructions

Procedure:

1. Wash your hands with soap and water
2. Open the test tube and place upright in cup
3. Open the sterile package that the swab is contained in and remove swab from sterile plastic package by touching only the plastic handle
4. Tilt you head slightly back as pictured
5. Gently place swab inside your nostril and using a gentle rotation, slowly push swab deeper in nostril until you feel slight resistance; less than 1 inch inside of your nose
6. When swab is in position, rotate the swab 2 times in a circular motion touching nasal walls and keep it in place for 5 seconds
7. After 5 seconds, remove swab from nose and place the side of swab which was in the nose DIRECTLY inside the test tube. DO NOT LAY THE SWAB DOWN OR ALLOW SWAB TO TOUCH ANYTHING ELSE
8. Break or cut off the handle of the swab and close the test tube
9. Seal test tube in bag and place sealed test tube in cup and put in refrigerator. Call health volunteer to schedule pick-up at
   ____

How to take a nasopharyngeal swab

Materials:

- Swab
- Test tube with Transport Media (TM)
- Test tube rack

Procedure:

1. Wash your hands with soap and water
2. Wear mask and gloves
3. Open the test tube and place upright in rack provided
4. Open the sterile package that the swab is contained in and remove swab from sterile plastic package by touching only the plastic handle
5. Tilt participant's head slightly back as pictured
6. Gently place swab inside participant's nostril and slowly push straight back without tilting upward until slight resistance is felt, as pictured below (distance from participants nostril to ear is approximately how deep the swab should be inserted). Do not force swab, if obstructions are met, try other nostril.
7. Hold swab in place for 5 seconds and slowly withdraw the swab while rotating it
8. Remove swab from nose and place the side of swab which was in the nose DIRECTLY inside the test tube. DO NOT LAY THE SWAB DOWN OR ALLOW SWAB TO TOUCH ANYTHING ELSE
9. Break off the handle of the swab and close the test tube
